# Highly Unsaturated Binuclear Butadiene Iron Carbonyls: Quintet Spin States, Perpendicular Structures, Agostic Hydrogen Atoms, and Iron-Iron Multiple Bonds

**DOI:** 10.3390/ijms12042216

**Published:** 2011-03-30

**Authors:** Yi Zeng, Shijian Wang, Hao Feng, Yaoming Xie, R. Bruce King

**Affiliations:** 1 School of Physics and Chemistry, Research Center for Advanced Computation, Xihua University, Chengdu, China; E-Mails: zengyi08@hotmail.com (Y.Z.); xhuwsj@163.com (S.W.); 2 Department of Chemistry and Center for Computational Chemistry; University of Georgia, Athens, GA 30602, USA; E-Mail: ymxie1@yahoo.com

**Keywords:** iron carbonyls, iron-iron bonding, agostic hydrogen atom, metal-olefin complexes

## Abstract

The highly unsaturated binuclear butadiene iron carbonyls (C_4_H_6_)_2_Fe_2_(CO)*_n_* (*n* = 2, 1) have been examined using density functional theory. For (C_4_H_6_)_2_Fe_2_(CO)*_n_* (*n* = 2, 1), both coaxial and perpendicular structures are found. The global minima of (C_4_H_6_)_2_Fe_2_(CO)*_n_* (*n* = 2, 1) are the perpendicular structures **2Q-1** and **1Q-1**, respectively, with 17- and 15-electron configurations for the iron atoms leading to quintet spin states. The Fe=Fe distance of 2.361 Å (M06-L) in the (C_4_H_6_)_2_Fe_2_(CO)_2_ structure **2Q-1** suggests a formal double bond. The Fe≡Fe bond distance in the (C_4_H_6_)_2_Fe_2_(CO) structure **1Q-1** is even shorter at 2.273 Å (M06-L), suggesting a triple bond. Higher energy (C_4_H_6_)_2_Fe_2_(CO)*_n_* (*n* = 2, 1) structures include structures in which a bridging butadiene ligand is bonded to one of the iron atoms as a tetrahapto ligand and to the other iron atom through two agostic hydrogen atoms from the end CH_2_ groups. Singlet (C_4_H_6_)_2_Fe_2_(CO) structures with formal Fe–Fe quadruple bonds of lengths ∼2.05 Å were also found but at very high energies (∼47 kcal/mol) relative to the global minimum.

## 1. Introduction

**Figure 1.**
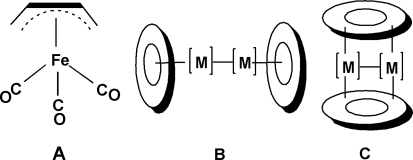
Structure of (η^4^-C_4_H_6_)Fe(CO)_3_ (**A**) and two general structure types for binuclear metal carbonyls, coaxial (**B**) and perpendicular (**C**).

The chemistry of metal carbonyl complexes of acyclic hydrocarbons dates back to the 1930 discovery by Reihlen *et al.* [1] of the mononuclear butadiene iron tricarbonyl complex, C_4_H_6_Fe(CO)_3_ by the reaction of butadiene with iron pentacarbonyl at elevated temperatures. The proposed tetrahapto bonding of the butadiene ligand to the Fe(CO)_3_ unit in this complex was confirmed in 1963 by Mills and Robinson [2] using X-ray crystallography at −40 °C (Figure 1A). In addition, in 1962 Murdoch and Weiss [3] used the reaction of butadiene with Fe_2_(CO)_9_ at room temperature to synthesize the tetracarbonyl (η^2^-C_4_H_6_)Fe(CO)_4_ in which only one of the two C═C double bonds of the butadiene ligand is bonded to the iron atom. An additional product from the latter reaction was the binuclear complex C_4_H_6_[Fe(CO)_4_]_2_ in which each C═C double bond of the butadiene ligand is bonded to a separate Fe(CO)_4_ unit with the iron atoms much too far apart for any kind of direct iron-iron bond. However, no binuclear butadiene iron carbonyl derivatives with short iron-iron distances suggesting iron-iron bonds have been synthesized. In order to assess the possibilities for binuclear iron carbonyl derivatives with iron-iron bonds we have performed a density functional theory (DFT) study on possible structures for (η^4^-C_4_H_6_)_2_Fe_2_(CO)*_n_* (*n* = 5, 4, 3), predicted to have structures with formal Fe–Fe single bonds, Fe═Fe double bonds, and Fe≡Fe triple bonds, respectively [4]. In general, the lowest energy structures for these (η^4^-C_4_H_6_)_2_Fe_2_(CO)*_n_* derivatives were found to be coaxial structures in which each metal atom is bonded to a single butadiene ligand (Figure 1B).

This paper reports a DFT study of the still more highly unsaturated (C_4_H_6_)_2_Fe_2_(CO)*_n_* (*n* = 2, 1) derivatives. Such systems are interesting since strict adherence to the 18-electron rule suggests that these highly unsaturated derivatives might provide examples of very short formal iron-iron quadruple and quintuple bonds. However, for such systems containing two or fewer carbonyl groups, alternative perpendicular structures are possible in which the butadiene ligands bridge the pair of iron atoms (Figure 1C). Structures of both types were found in this work. Quintet spin state structures were found to be the lowest energy structures for both (C_4_H_6_)_2_Fe_2_(CO)_2_ and (C_4_H_6_)_2_Fe_2_(CO). In addition, interesting structures were found with agostic hydrogen atoms bridging Fe–C bonds.

## 2. Theoretical Methods

Density functional theory (DFT) including electron correlation effects has been shown to be a powerful and effective computational tool in organotransition metal chemistry [5–19]. Three DFT methods were used for this work. The first functional was BP86, which combines Becke’s 1988 exchange functional (B) with Perdew’s 1986 gradient corrected correlation functional method (P86), and usually provides better vibrational frequencies [20,21]. The second DFT method was B3LYP, which is the hybrid HF/DFT functional using the combination of the three-parameter Becke functional (B3) [22] with the Lee-Yang-Parr (LYP) generalized gradient correlation functional [23]. The third was a hybrid meta-GGA DFT method, M06-L, developed by Truhlar’s group [24]. Recently Truhlar’s group made much progress to the development of improved exchange-correlation functionals that are essential for expanding the applicability of Kohn–Sham DFT, such as the M06 suite. Thus M06-L was constructed using three strategies: constraint satisfaction, modeling the exchange-correlation hole, and empirical fits. They concluded that M06-L is one of the best functionals for the study of organometallic and inorganic thermochemistry, and is the best functional for transition metal energetics. In comparing the first two DFT methods Reiher and collaborators found that B3LYP always overestimates the energy of high-spin states and BP86 overestimates the energies of low-spin states for a series of the Fe(II)-S complexes [25]. In the present study, we found that the M06-L method predicts an intermediate energy difference, anticipated to be closer to the experimental. We therefore adopt the energy order predicted by the M06-L method, but list the BP86 and B3LYP results in the Supporting Information.

Basis sets have been chosen to provide continuity with a body of existing research on organometallic compounds. Fortunately, DFT methods are far less basis set sensitive than higher-level methods such as coupled cluster theory. In this work, the double-ζ plus polarization (DZP) basis sets used for C and O add one set of pure spherical harmonic d functions with orbital exponents α_d_(C) = 0.75 and α_d_(O) = 0.85 to the Huzinaga Dunning standard contracted DZ sets and are denoted as (9s5p1d/4s2p1d) [26,27]. For H, a set of p polarization functions α_p_(H) = 0.75 is added to the Huzinaga Dunning DZ sets. For Fe, in our loosely contracted DZP basis set, the Wachters’ primitive set [28] is used after being augmented by two sets of p functions and one set of d functions and then contracted using the method of Hood, Pitzer, and Schaefer [29]. This basis set is denoted as (14s11p6d/10s8p3d).

The geometries of the structures were fully optimized using the Gaussian09 program [30] with the three selected DFT methods and with the indicated DZP basis set. The vibrational frequencies were determined by evaluating analytically the second derivatives of the energy with respect to the nuclear coordinates at the same levels. The corresponding infrared intensities were also evaluated analytically. The fine grid (75, 302) was the default for evaluating integrals numerically, and the tight designation was the default for the energy convergence, as well as the tight option for the geometry optimizations. In some cases, the finer grid (120, 974) was used for investigating small imaginary vibrational frequencies. Natural bond orbital (NBO) analyses [31] used the DZP BP86 method with the NBO 3.1 version attached in the Gaussian03 program.

**Figure 2.**
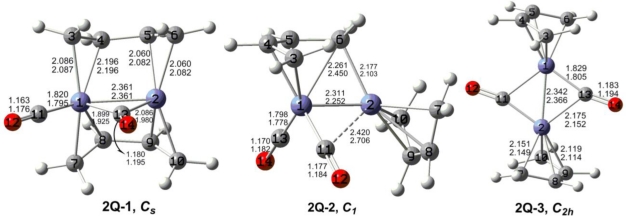
Quintet structures for (C_4_H_6_)_2_Fe_2_(CO)_2_. The upper bond distances were obtained by the M06-L method and the lower bond distances by the BP86 method.

**Figure 3.**
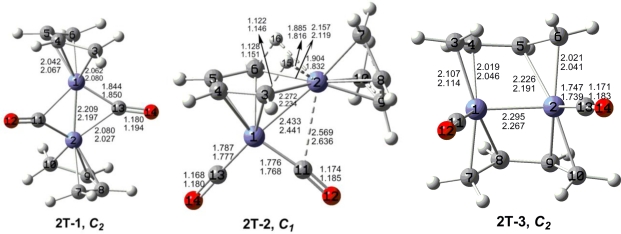
Triplet structures for (C_4_H_6_)_2_Fe_2_(CO)_2_. The upper bond distances were obtained by the M06-L method and the lower bond distances by the BP86 method.

**Figure 4.**
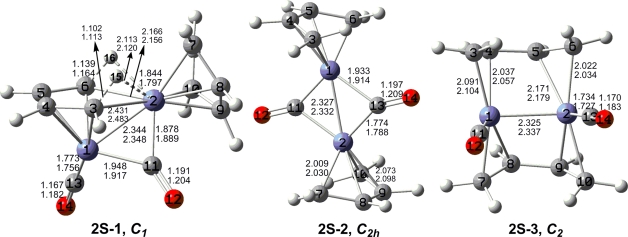
Singlet structures for (C_4_H_6_)_2_Fe_2_(CO)_2_. The upper bond distances were obtained by the M06-L method and the lower bond distances by the BP86 method.

**Figure 5.**
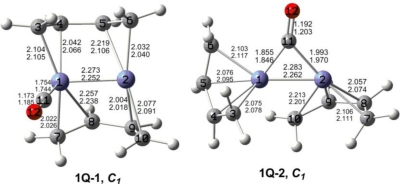
Quintet structures for (C_4_H_6_)_2_Fe_2_(CO). The upper bond distances were obtained by the M06-L method and the lower bond distances by the BP86 method.

**Figure 6.**
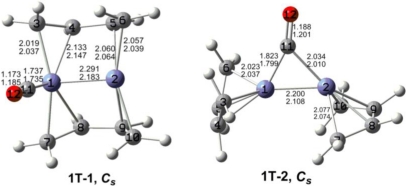
Triplet structures for (C_4_H_6_)_2_Fe_2_(CO). The upper bond distances were obtained by the M06-L method and the lower bond distances by the BP86 method.

**Figure 7.**
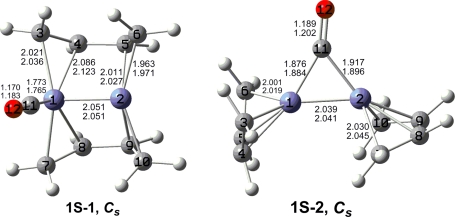
Singlet structures for (C_4_H_6_)_2_Fe_2_(CO). The upper bond distances were obtained by the M06-L method and the lower bond distances by the BP86 method.

The optimized (C_4_H_6_)_2_Fe_2_(CO)*_n_* (*n* = 2, 1) structures are depicted in Figures 2 to 7. In these figures, the upper and lower distances were obtained by the M06-L and BP86 method, respectively. The structures are designated as **nX-m**, where **n** stands for the number of CO groups, **X** designates the spin state using **S** for singlets, **T** for triplets and **Q** for quintets, and **m** orders the structures according to their relative energies. Note that, although the singlets, the triplets and the quintets are discussed in separate sections, the relative energies are considered together on the basis of the number of carbonyls. The M06-L method appears to predict the better singlet-triplet splittings.

The relative energies corrected for zero-point energies are listed in the Supporting Information, where computed enthalpies and free energies are also given. These relative free energies agree within 2 kcal/mol with the relative electronic energies.

## 3. Results and Discussion

### 3.1. (C_4_H_6_)_2_Fe_2_(CO)_2_

Three types of stationary points, namely, coaxial structures, perpendicular structures, and deformed coaxial structures, have been found for (C_4_H_6_)_2_Fe_2_(CO)_2_. The global minimum of the (C_4_H_6_)_2_Fe_2_(CO)_2_ is the quintet structure **2Q-1** according to the relative energies listed in Tables 1, 2, and 3.

#### 3.1.1. Quintet (C_4_H_6_)_2_Fe_2_(CO)_2_ Structures

Three quintet structures were found for (C_4_H_6_)_2_Fe_2_(CO)_2_ (Figure 2 and Table 1). The perpendicular structure **2Q-1** with a bridging CO group and two bridging butadiene ligands is the global minimum. The Fe=Fe distance in **2Q-1** is predicted by M06-L to be 2.361 Å. This can be interpreted as a formal Fe=Fe double bond to give one iron atom a 17-electron configuration and the other iron atom a 15-electron configuration. This can correspond to a quintet spin state.

A deformed coaxial (C_4_H_6_)_2_Fe_2_(CO)_2_ structure **2Q-2** with one terminal CO group, one semibridging carbonyl group, and one bridging butadiene ligand is predicted to lie only 1.5 kcal/mol (M06-L) in energy above the global minimum **2Q-1** (Figure 2 and Table 1). The bridging butadiene ligand in **2Q-2** is bonded as a trihapto ligand to one iron atom and as a monohapto ligand to the other iron atom. The Fe=Fe bond distance in **2Q-2** of 2.311 Å (M06-L) is close to that in **2Q-1** and can likewise be interpreted as a formal double bond. Again this gives one iron atom a 15-electron configuration and the other iron atom a 17-electron configuration, which can correspond to a quintet spin state.

The coaxial (C_4_H_6_)_2_Fe_2_(CO)_2_ structure **2Q-3** has two bridging carbonyl groups and lies 8.0 kcal/mol in energy above **2Q-1**. The Fe=Fe bond distance in **2Q-3** is 2.342 Å (M06-L), which is similar to those in **2Q-1** and **2Q-2** and thus can correspond to a formal double bond. This gives each iron atom in **2Q-3** a 16-electron configuration, which can correspond to a quintet spin state.

#### 3.1.2. Triplet (C_4_H_6_)_2_Fe_2_(CO)_2_ Structures

Three triplet structures were found for (C_4_H_6_)_2_Fe_2_(CO)_2_ (Figure 3 and Table 2). The lowest energy of these triplet structures, namely **2T-1** lying 6.8 kcal/mol in energy above the quintet global minimum **2Q-1**, is a coaxial structure with two bridging carbonyl groups and two terminal butadiene ligands. The Fe≡Fe bond distance of 2.209 Å (M06-L) in **2T-1** is ∼0.1 Å shorter than that in the similar quintet spin state structure **2Q-3** and thus can be interpreted as a formal triple bond. This gives each iron atom in **2T-1** a 17-electron configuration consistent with a binuclear triplet.

The next triplet (C_4_H_6_)_2_Fe_2_(CO)_2_ structure, namely **2T-2**, lying 8.4 kcal/mol above the quintet global minimum **2Q-1**, is a deformed coaxial structure with one bridging butadiene ligand, one terminal butadiene ligand, and two terminal carbonyl groups (Figure 3 and Table 2). The bridging butadiene ligand in **2T-2** is bonded to one of the iron atoms as a tetrahapto ligand. In addition, the two terminal hydrogen atoms of this butadiene ligand are agostic hydrogen atoms bonding to the other iron atom through C-H-Fe bridging units with Fe–C distances of ∼2.2 Å and Fe–H distances of ∼1.9 Å. These C–H–Fe units are predicted to exhibit abnormally low ν(C–H) frequencies of 2515 and 2561 cm^–1^. The Fe–Fe distance in **2T-2** is relatively long at 2.433 Å (M06-L) and can thus be considered as a formal single bond. This gives each iron atom in **2T-2** the 17-electron configuration for a binuclear triplet.

The perpendicular triplet (C_4_H_6_)_2_Fe_2_(CO)_2_ structure **2T-3** has two bridging butadiene ligands and two terminal carbonyl groups and lies 12.3 kcal/mol (M06-L) in energy above the global minimum **2Q-1** (Figure 3 and Table 2). The Fe≡Fe distance of 2.295 Å (M06-L) in **2T-3** can correspond to a formal triple bond to give each iron atom a 17-electron configuration for a binuclear triplet.

#### 3.1.3. Singlet (C_4_H_6_)_2_Fe_2_(CO)_2_ Structures

Three distinct singlet structures are obtained for (C_4_H_6_)_2_Fe_2_(CO)_2_. However, they are all high energy structures lying from 19 to 30 kcal/mol above the **2Q-1** global minimum (Figure 4 and Table 3). The lowest energy singlet (C_4_H_6_)_2_Fe_2_(CO)_2_ structure **2S-1**, lying 19.1 kcal/mol above **2Q-1**, is very similar to the triplet structure **2T-2**. Thus structure **2S-1** has a bridging butadiene ligand connected to one iron atom as a tetrahapto ligand and to the other atom through two non-equivalent C–H–Fe bridges with Fe–C distances of 2.166 and 2.431 Å and Fe–H distances of 1.844 and 2.113 Å (M06-L). The strikingly lower ν(C–H) vibrational frequencies 2392 cm^−1^ and 2912 cm^−1^ (BP86) and the longer C–H bond distances involving these agostic hydrogens confirm the weaker C–H bonds. One of the carbonyl groups in **2S-1** is a nearly symmetrical bridging carbonyl group with Fe–C distances of 1.878 and 1.948 Å (M06-L). The remaining carbonyl group and butadiene ligand in **2S-1** are both terminal groups. The predicted Fe=Fe bond length of 2.344 Å (M06-L) in **2S-1** is ∼0.1 Å longer than the formal Fe–Fe single bond in **2T-2** and thus can be considered to be a formal double bond, thereby giving both iron atoms the favored 18-electron configuration in **2S-1**.

The *C*_2_*_h_* coaxial singlet (C_4_H_6_)_2_Fe_2_(CO)_2_ structure **2S-2** with two bridging carbonyl groups and terminal butadiene ligands is predicted to lie 23.9 kcal/mol (M06-L) in energy above the global minimum **2Q-1** (Figure 4 and Table 3). The Fe=Fe bond distance of 2.327 Å (M06-L) in **2S-2** is similar to that in **2S-1** and thus can be assigned to a formal double bond. This gives each iron atom in **2S-2** a 16-electron configuration, which can relate to a binuclear singlet.

The *C*_2_ singlet (C_4_H_6_)_2_Fe_2_(CO)_2_ structure **2S-3** is a perpendicular structure lying 30.0 kcal/mol (M06-L) above the **2Q-1** global minimum (Figure 4 and Table 3). Both butadiene ligands in **2S-3** are bridging ligands and both carbonyl ligands in **2S-3** are terminal ligands. The Fe=Fe distance of 2.325 Å in **2S-3** is similar to those in **2S-1** and **2S-2** and likewise can correspond to a formal double bond. This gives each iron atom in **2S-3** a 16-electron configuration suggesting a vacant coordination site on each iron atom. This is consistent with the geometry of the carbonyl groups in **2S-3**.

The overall energy order for (C_4_H_6_)_2_Fe_2_(CO)_2_ structures investigated in this section is **2Q-1** < **2Q-2** < **2T-1** < **2Q-3** ∼ **2T-2** < **2T-3** < **2S-1** < **2S-2** < **2S-3** by M06-L. This suggests that (C_4_H_6_)_2_Fe_2_(CO)_2_ prefers high spin state structures. The energy gaps between the HOMOs and LUMOs in Tables 1, 2 and 3 for **2S-1** → **2T-2** → **2Q-2** are 1.01, 1.86 and 1.97 eV, respectively, which increase monotonically with the increase of the spin multiplicity. The same trend is also found for **2S-2** → **2T-1** → **2Q-3** and **2S-3** → **2T-3** → **2Q-1.**

### 3.2. (C_4_H_6_)_2_Fe_2_(CO)

Two types of stationary points for (C_4_H_6_)_2_Fe_2_(CO), namely, coaxial and perpendicular structures, are shown in Figures 5, 6 and 7. The global minimum of the (C_4_H_6_)_2_Fe_2_(CO) is the quintet structure **1Q-1** according to the relative energies listed in Tables 4, 5, and 6.

#### 3.2.1. Quintet (C_4_H_6_)_2_Fe_2_(CO) Structures

Two quintet structures were found for (C_4_H_6_)_2_Fe_2_(CO) (Figure 5 and Table 4). The perpendicular (C_4_H_6_)_2_Fe_2_(CO) global minimum **1Q-1** has two bridging butadiene ligands and a terminal carbonyl group. The Fe≡Fe distance of 2.273 Å (M06-L) can correspond to a formal triple bond thereby giving the iron atom bearing the carbonyl group a 17-electron configuration but the other iron atom only a 15-electron configuration. This is consistent with a quintet spin multiplicity.

The other quintet (C_4_H_6_)_2_Fe_2_(CO) structure **1Q-2** has a bridging CO group and lies 12.9 kcal/mol above the global minimum **1Q-1** (Figure 5 and Table 4). The Fe≡Fe distance of 2.283 Å (M06-L) in **1Q-2** is similar to that in **1Q-1** and likewise can correspond to a formal triple bond. This gives both iron atoms a 16-electron configuration, which can correspond to a binuclear quintet spin state.

#### 3.2.2. Triplet (C_4_H_6_)_2_Fe_2_(CO) Structures

Two triplet (C_4_H_6_)_2_Fe_2_(CO) structures were found to lie 12 to 24 kcal/mol in energy above the quintet global minimum **1Q-1** using the M06-L method (Figure 6 and Table 5). The perpendicular triplet (C_4_H_6_)_2_Fe_2_(CO) structure **1T-1**, lying 11.6 kcal/mol above **1Q-1**, has two bridging butadiene ligands and a terminal carbonyl group. Considerable spin contamination was found for **1T-1**. Thus the spin expectation value 〈*S*^2^〉 = 2.82 for **1T-1** as compared with the ideal 2.0. Indeed, a more stable quintet (C_4_H_6_)_2_Fe_2_(CO) structure **1Q-1** is found with an 〈*S*^2^〉 value within 10% of the ideal 6.0. The Fe≡Fe distance of 2.291 Å (M06-L) is very similar to the Fe≡Fe triple bond distances in the quintet structures **1Q-1** and **1Q-2** and thus likewise can correspond to a formal triple bond. This gives the iron atom in **1T-1** bearing the carbonyl group a 17-electron configuration but the other iron atom only a 15-electron configuration. This can correspond to a binuclear triplet with a vacant coordination position on the iron atom with only a 15-electron configuration. The *C_s_* coaxial triplet (C_4_H_6_)_2_Fe_2_(CO) structure **1T-2**, lying 24.2 kcal/mol (M06-L) above **1Q-1** has a bridging carbonyl group but terminal butadiene ligands. The predicted Fe≡Fe distance of 2.200 Å (M06-L) is similar to the Fe≡Fe distances in the other quintet and triplet (C_4_H_6_)_2_Fe_2_(CO) structures and likewise can correspond to a formal triple bond. This gives each iron atom in **1T-2** a 16-electron configuration.

#### 3.2.3. Singlet (C_4_H_6_)_2_Fe_2_(CO) Structures

Two singlet low-lying (C_4_H_6_)_2_Fe_2_(CO) structures were found (Figure 7 and Table 6) but at very high energies relative to the corresponding quintet and triplet structures. Thus the *C_s_* perpendicular singlet structure **1S-1** lies 46.7 kcal/mol above the quintet global minimum **1Q-1**. Structure **1S-1** has a terminal carbonyl group and bridging butadiene ligands. The Fe–Fe distance in **1S-1** of 2.051 Å is ∼0.2 Å shorter than the Fe≡Fe distances in the quintet and triplet (C_4_H_6_)_2_Fe_2_(CO) structures and thus can correspond to a formal quadruple bond. This gives the iron atom in **1S-1** bearing the carbonyl group the favored 18-electron configuration but the other iron atom only a 16-electron configuration.

The *C_s_* singlet coaxial (C_4_H_6_)_2_Fe_2_(CO) structure **1S-2** is also a high energy structure, lying 47.4 kcal/mol above the global minimum **1S-1** (Figure 7 and Table 6). Structure **1S-2** has terminal butadiene ligands and a bridging carbonyl group. The very short Fe–Fe distance of 2.039 Å (M06-L) in **1S-2** is similar to that in **1S-1** and thus likewise can correspond to a formal quadruple bond. This gives one iron atom in **1S-2** the favored 18-electron configuration but the other iron atom only a 16-electron configuration. This asymmetry in the electron count on the iron atoms in **1S-2** is reflected in a different arrangement of the η^4^-butadiene ligands on each iron atom. Thus, the “right” iron atom in **1S-2** as depicted in Figure 7 appears to have a vacant coordination site and thus can correspond to the iron atom with a 16-electron configuration.

The overall energy order for the (C_4_H_6_)_2_Fe_2_(CO) structures investigated in this section is **1Q-1** < **1T-1** < **1Q-2** < **1T-2** < **1S-1**∼**1S-2** (M06-L). Thus the higher spin states are energetically favored. The energy gaps between the HOMOs and LUMOs in Tables 4, 5, and 6 for **1S-1** → **1T-1** → **1Q-1** are 0.39, 1.39 and 2.31 eV, respectively, and that for **1S-2** → **1T-2** → **1Q-2** are 0.61, 1.93 and 1.72 eV, respectively.

### 3.3. NBO Analysis

The natural charges on the iron atoms and the Wiberg Bond Indices (WBIs) for the iron-iron bonds are listed in Table 7 along with the Fe–Fe distances, the iron electronic configurations, and formal iron-iron bond orders. For the less unsaturated binuclear butadiene iron carbonyls (C_4_H_6_)_2_Fe_2_(CO)*_n_* (*n* = 7, 6, 5, 4, 3) studied previously [4] the natural charges on the iron atoms correlate mainly with the numbers of carbonyl groups on the iron atoms and the WBIs correlated with the formal iron-iron bond orders. However, the much greater variety of iron electronic configurations and spin states encountered in the highly unsaturated (C_4_H_6_)_2_Fe_2_(CO)*_n_* (*n* = 2, 1) structures reported in this paper make the interpretations much less clear. In this connection, iron atoms bonded to two CO groups were found to be essentially neutral. In most cases iron atoms bonded to only a single CO group (or half of two bridging carbonyl groups) have natural positive charges in the range 0.13 to 0.25. Carbonyl-free iron atoms are even more positive but their natural positive charges span a wide range from 0.28 to 0.83. Thus the previously observed [4] general trend of increasing natural negative charges on the iron atoms with increasing number of carbonyl ligands is also found here. However, other factors besides the number of carbonyl groups also affect significantly the natural charges on the iron atoms.

The WBIs for the iron-iron bonds in the (C_4_H_6_)_2_Fe_2_(CO)*_n_* (*n* = 2, 1) structures also showed the expected correlation of increased WBI with an increase in formal bond order (Table 7). However, the ranges of WBIs for a given formal iron-iron bond order are relatively broad indicating the significant influence of other factors. The one example of an Fe–Fe single bond in **2T-2** has WBI of 0.19. Most of the Fe=Fe double bonds have WBIs in the range 0.26 to 0.43. However, there are some unusually high WBIs for apparent Fe=Fe double bonds including 0.59 for the doubly bridged coaxial (C_4_H_6_)_2_Fe_2_(CO)_2_ structure **2S-2** and the very high value of 0.93 for the perpendicular (C_4_H_6_)_2_Fe_2_(CO)_2_ structure **2S-3**. Interpreting the iron-iron bonds in these two structures as formal quadruple bonds would rationalize these significantly higher WBIs and give both iron atoms the favorable 18-electron configurations but would be inconsistent with the iron-iron distances of ∼2.3 Å. The formal Fe≡Fe triple bonds in the (C_4_H_6_)_2_Fe_2_(CO)*_n_* (*n* = 2, 1) structures exhibit WBIs in the broad range 0.38 to 0.81. The two examples of formal Fe–Fe quadruple bonds, namely the Fe-Fe bonds in the singlet (C_4_H_6_)_2_Fe_2_(CO) structures **1S-1** and **1S-2**, exhibit by far the highest WBIs at 1.30 and 1.42, respectively, consistent with the high formal bond orders.

### 3.4. Vibrational Frequencies

Table 8 exhibits the ν(CO) frequencies and their infrared intensities for all of the (C_4_H_6_)_2_Fe_2_(CO)*_n_* (*n* = 2, 1) structures, evaluated using the BP86 method, which has been shown to be a reliable predictor of such ν(CO) frequencies. The terminal ν(CO) frequencies fall in the range from 1908 to 1962 cm^−1^ whereas the bridging ν(CO) frequencies are significantly lower falling in the range from 1761 to 1858 cm^−1^ (Table 8). The significantly lower ν(CO) frequencies for bridging relative to terminal carbonyls is well-established and is consistent with the lower effective C–O bond order for bridging relative to terminal carbonyl groups in a given type of metal carbonyl structure.

### 3.5. Thermochemistry

In order to check the potential experimental accessibility of the title compounds, we examined the following energies:

1. The dissociation energies of carbonyl groups from (C_4_H_6_)_2_Fe_2_(CO)_2_, namely:
  $${\left({\text{C}}_{4}{\text{H}}_{6}\right)}_{2}{\text{Fe}}_{2}{\left(\text{CO}\right)}_{2}\to {\left({\text{C}}_{4}{\text{H}}_{6}\right)}_{2}{\text{Fe}}_{2}(\text{CO})+\text{CO}$$
2. The energies of the following disproportionation reaction:
  $$2{\left({\text{C}}_{4}{\text{H}}_{6}\right)}_{2}{\text{Fe}}_{2}{\left(\text{CO}\right)}_{2}\to {\left({\text{C}}_{4}{\text{H}}_{6}\right)}_{2}{\text{Fe}}_{2}{\left(\text{CO}\right)}_{3}+{\left({\text{C}}_{4}{\text{H}}_{6}\right)}_{2}{\text{Fe}}_{2}(\text{CO})$$
3. The fragmentation energies of the binuclear (C_4_H_6_)_2_Fe_2_(CO)_2_ to mononuclear fragments by the following reaction:
  $${\left({\text{C}}_{4}{\text{H}}_{6}\right)}_{2}{\text{Fe}}_{2}{\left(\text{CO}\right)}_{2}\to 2{\text{C}}_{4}{\text{H}}_{6}\text{Fe}(\text{CO})$$

Table 9 lists the energies and corresponding free energies for the above reactions taking the energies of the structures (C_4_H_6_)_2_Fe_2_(CO)_3_ and C_4_H_6_Fe(CO) from ref. [4]. The predicted energy for loss of a single carbonyl group from (C_4_H_6_)_2_Fe_2_(CO)_2_ is large, namely ∼30 kcal/mol as well as a free energy of ∼20 kcal/mol. The disproportionation of (C_4_H_6_)_2_Fe_2_(CO)_2_ to give (C_4_H_6_)_2_Fe_2_(CO)_3_ and (C_4_H_6_)_2_Fe_2_(CO) using the BP86 method is exothermic, while it is endothermic using M06-L and B3LYP methods by 3.4 and 15.6 kcal/mol, respectively. The corresponding free energies reveal a similar trend. The predicted fragmentation energies of (C_4_H_6_)_2_Fe_2_(CO)_2_ to mononuclear C_4_H_6_Fe(CO) is significantly larger, namely ∼60 kcal/mol by any of the three methods.

## 4. Conclusions

Unsaturation in binuclear metal carbonyl derivatives can lead to metal-metal multiple bonding, four-electron donor bridging carbonyl groups, and/or metal electronic configurations less than the favorable 18-electron configurations. None of the highly unsaturated (C_4_H_6_)_2_Fe_2_(CO)*_n_* (*n* = 2, 1) structures found in this work has a four-electron donor bridging carbonyl group. Instead the lowest energy (C_4_H_6_)_2_Fe_2_(CO)*_n_* (*n* = 2, 1) structures are perpendicular structures having iron atoms with 15- and 17-electron configurations. This leads to quintet spin states in addition to iron-iron multiple bonds of formal order two for (C_4_H_6_)_2_Fe_2_(CO)_2_ and three for (C_4_H_6_)_2_Fe_2_(CO). In addition, agostic hydrogen atoms forming C-H-Fe bridges are seen to be a feature of (C_4_H_6_)_2_Fe_2_(CO)*_n_* (*n* = 2, 1) structures, albeit not the lowest energy such structures. In such structures a butadiene ligand is bonded to one of the iron atoms as a tetrahapto ligand and to the other iron atom through two C–H–Fe units from the end CH_2_ groups with agostic hydrogen atoms bridging iron-carbon bonds. Singlet (C_4_H_6_)_2_Fe_2_(CO) structures with formal Fe–Fe quadruple bonds of lengths ∼2.05 Å were also found but at very high energies (∼47 kcal/mol) relative to the global minimum.

## Figures and Tables

**Table 1.** Fe–Fe distances (Å), HOMO-LUMO energies (*E*, in hartree), HOMO-LUMO gaps (in eV), total energies (*E*, in hartree), relative energies (Δ*E*, in kcal/mol, numbers of imaginary frequencies (Nimag) and spin expectation values 〈*S*^2^〉 for the quintet (C_4_H_6_)_2_Fe_2_(CO)_2_ structures with the M06-L method.

		**2Q-1** (*C_s_*)	**2Q-2** (*C*_1_)	**2Q-3** (*C*_2_*_h_*)
M06-L	Fe–Fe	2.361	2.311	2.342
	HOMO(α)	−0.17183	−0.17530	−0.17208
	LUMO(α)	−0.09384	−0.10290	−0.11149
	gap/eV	2.12	1.97	1.65
	*E*	−3066.14608	−3066.14371	−3066.13340
	Δ*E*	0.0	1.5	8.0
	Nimag	none	none	none
	〈*S*^2^〉	6.30	6.31	6.63

**Table 2.** Fe–Fe distances (Å), HOMO-LUMO energies (*E*, in hartree), HOMO-LUMO gaps (in eV), total energies (*E*, in hartree), relative energies (Δ*E*, in kcal/mol), numbers of imaginary frequencies (Nimag) and spin expectation values 〈*S*^2^〉 for the triplet (C_4_H_6_)_2_Fe_2_(CO)_2_ structures with the M06-L method.

		**2T-1** (*C*_2_)	**2T-2** (*C*_1_)	**2T-3** (*C*_2_)
M06-L	Fe-Fe	2.209	2.433	2.295
	HOMO(α)	−0.19453	−0.17557	−0.15910
	LUMO(α)	−0.12453	−0.10739	−0.10465
	gap/eV	1.90	1.86	1.48
	*E*	−3066.13525	−3066.13271	−3066.1265
	Δ*E*	6.8	8.4	12.3
	Nimag	none	none	none
	〈*S*^2^〉	2.20	2.12	2.21

**Table 3.** Fe–Fe distances (Å), HOMO-LUMO energies (*E*, in hartree), HOMO-LUMO gaps (in eV), total energies (*E*, in hartree), relative energies (Δ*E*, in kcal/mol) and numbers of imaginary frequencies (Nimag) for the singlet (C_4_H_6_)_2_Fe_2_(CO)_2_ structures with the M06-L method.

		**2S-1** (*C*_1_)	**2S-2** (*C*_2_*_h_*)	**2S-3** (*C*_2_)
M06-L	Fe-Fe	2.344	2.327	2.325
	HOMO	−0.15788	−0.17415	−0.13105
	LUMO	−0.12068	−0.13219	−0.11009
	gap/eV	1.01	1.14	0.57
	*E*	−3066.11570	−3066.10797	−3066.09822
	Δ*E*	19.1	23.9	30.0
	Nimag	none	none	none

**Table 4.** Fe–Fe distances (Å), HOMO-LUMO energies (*E*, in hartree), HOMO-LUMO gaps (in eV), total energies (*E*, in hartree), relative energies (Δ*E*, in kcal/mol, numbers of imaginary frequencies (Nimag) and spin expectation values 〈*S*^2^〉 for the quintet (C_4_H_6_)_2_Fe_2_(CO) structures with the M06-L method.

		**1Q-1** (*C*_1_)	**1Q-2** (*C*_1_)
M06-L	Fe-Fe	2.290	2.283
	HOMO(α)	−0.16350	−0.17107
	LUMO(α)	−0.07858	−0.10795
	gap/eV	2.31	1.72
	*E*	−2952.77563	−2952.75505
	Δ*E*	0.0	12.9
	Nimag	none	none
	〈*S*^2^〉	6.32	6.40

**Table 5.** Fe–Fe distances (Å), HOMO-LUMO energies (*E*, in hartree), HOMO-LUMO gaps (in eV), total energies (*E*, in hartree), relative energies (Δ*E*, in kcal/mol, numbers of imaginary frequencies (Nimag) and spin expectation values 〈*S*^2^〉 for the triplet (C_4_H_6_)_2_Fe_2_(CO) structures using the M06-L method.

		**1T-1** (*C_s_*)	**1T-2** (*C_s_*)
M06-L	Fe–Fe	2.291	2.200
	HOMO(α)	−0.15400	−0.164149
	LUMO(α)	−0.102794	−0.093119
	gap/eV	1.39	1.93
	*E*	−2952.75716	−2952.73705
	Δ*E*	11.6	24.2
	Nimag	none	none
	〈*S*^2^〉	2.82	2.22

**Table 6.** Fe–Fe distances (Å), HOMO-LUMO energies (*E*, in hartree), HOMO-LUMO gaps (in eV), total energies (*E*, in hartree), relative energies (*ΔE*, in kcal/mol, and numbers of imaginary frequencies (Nimag) for the singlet (C_4_H_6_)_2_Fe_2_(CO) structures with the M06-L method.

		**1S-1** (*C_s_*)	**1S-2** (*C_s_*)
M06-L	Fe–Fe	2.051	2.039
	HOMO	−0.12987	−0.14810
	LUMO	−0.11542	−0.12583
	gap/eV	0.39	0.61
	*E*	−2952.70120	−2952.70002
	Δ*E*	46.7	47.4
	Nimag	none	none

**Table 7.** Fe–Fe distances, NPA natural charges, iron electron configurations, traditional formal Fe–Fe bond orders and Wiberg bond indices (WBIs) for the (C_4_H_6_)_2_Fe_2_(CO)*_n_* (*n* = 2, 1) structures using the BP86 method. Global minima structures are in **bold** type.

**(η^4^-C_4_H_6_)_2_Fe_2_(CO)_n_**			**Fe–Fe Distance**	**Fe Natural Charge**		**Fe Electron Configuration**		**Formal Bond Order**	**Wiberg Bond Index**

				**Fe1**	**Fe2**	**Fe1**	**Fe2**		
*n* = 2	**2Q-1**	***Cs***	**2.361**	**0.164**	**0.685**	**17**	**15**	**2**	**0.26**
	2Q-2	*C*_1_	2.252	−0.066	0.834	17	15	2	0.45
	2Q-3	*C*_2_*_h_*	2.366	0.467	0.467	16	16	2	0.38

	2T-1	*C*_i_	2.197	0.251	0.251	17	17	3	0.52
	2T-2	*C*_1_	2.441	0.023	0.335	17	17	1	0.19
	2T-3	*C*_2_	2.267	0.157	0.157	17	17	3	0.52

	2S-1	*C*_1_	2.348	0.095	0.097	18	18	2	0.26
	2S-2	*C*_2_*_h_*	2.332	0.121	0.121	16	16	2	0.59
	2S-3	*C*_2_	2.337	0.206	0.206	16	16	2	0.93

*n* = 1	**1Q-1**	***C*_1_**	**2.252**	**0.187**	**0.742**	**17**	**15**	**3**	**0.52**
	1Q-2	*C*_1_	2.262	0.371	0.663	16	16	3	0.38

	1T-1	*Cs*	2.183	0.177	0.682	17	15	3	0.86
	1T-2	*Cs*	2.108	0.194	0.503	16	16	3	0.81

	1S-1	*Cs*	2.051	0.131	0.467	18	16	4	1.30
	1S-2	*Cs*	2.041	0.278	0.385	18	16	4	1.42

**Table 8.** The ν(CO) frequencies (cm^−1^) and their infrared intensities (km/mol, in parentheses) for (C_4_H_6_)_2_Fe_2_(CO)*_n_* (*n* = 2, 1) structures as determined by the BP86 method. Bridging ν(CO) frequencies are in **bold** type.

(C_4_H_6_)_2_Fe_2_(CO)_2_	**2S-1** (*C*_2_*_h_*)	**1793 (a**, **491)**, 1942(a, 1037)
	**2S-2** (*C*_1_)	**1761 (b_u_**, **883)**, **1783 (a_g_**, **0)**
	**2S-3** (*C*_2_)	1925 (b, 424), 1955 (a,1040)
	**2T-1** (*C*_2_)	**1842 (b**, **1078)**, **1858 (a**, **65)**
	**2T-2** (*C*_1_)	1908 (a, 485), 1957 (a,1296)
	**2T-3** (*C*_2_)	1925 (a, 656), 1950 (a, 1043)
	**2Q-1** (*C_s_*)	**1815 (a’**, **454)**, 1962 (a’, 1045)
	**2Q-2** (*C*_1_)	1912 (a, 798), 1948 (a, 706)
	**2Q-3** (*C*_2_*_h_*)	**1842 (b_u_**, **1002)**, **1858 (a_g_**, **0)**

(C_4_H_6_)_2_Fe_2_(CO)	**1S-1** (*C_s_*)	1933 (a’, 901)
	**1S-2** (*C_s_*)	**1826 (a’**, **716)**
	**1T-1** (*C_s_*)	1929 (a’, 857)
	**1T-2** (*C_s_*)	**1835 (a’**, **681)**
	**1Q-1** (*C*_1_)	1925 (a, 870)
	**1Q-2** (*C*_1_)	**1802 (a**, **666)**

**Table 9.** Dissociation energy for removal of one carbonyl group from (C_4_H_6_)_2_Fe_2_(CO)_2_, disproportionation energy for (C_4_H_6_)_2_Fe_2_(CO)_2_, and dissociation energy for (C_4_H_6_)_2_Fe_2_(CO)_2_ → 2C_4_H_6_Fe(CO). The corresponding free energies are in *italics* (kcal/mol).

		**BP86**	**M06-L**	**B3LYP**
(C_4_H_6_)_2_Fe_2_(CO)_2_(**2Q-1**) → (C_4_H_6_)_2_Fe_2_(CO)(**1Q-1**) +CO	E	34.1	33.9	30.0
	G	*22.1*	*22.5*	*15.6*
2(C_4_H_6_)_2_Fe_2_(CO)_2_(**2Q-1**) → (C_4_H_6_)_2_Fe_2_(CO)_3_(**3S-1**) + (C_4_H_6_)_2_Fe_2_(CO) (**1Q-1**)	E	−13.9	3.4	15.6
	G	−*10.5*	*9.2*	*18.0*
(C_4_H_6_)_2_Fe_2_(CO)_2_(**2Q-1**) → 2C_4_H_6_Fe(CO)	E	59.1	73.7	59.3
	G	*46.6*	*62.6*	*47.6*

## Supporting Information

Tables S1 to S12: Fe–Fe distances (Å), total energies (*E*, in hartree), relative energies (*E*), zero-point energies (ZPE), enthalpies (Δ*H*), free energies (Δ*G*, in kcal/mol) and numbers of imaginary frequencies (Nimag) for (C_4_H_6_)_2_Fe_2_(CO)*_n_* (*n* = 2, 1) structures at M06-L, BP86 and B3LYP; Tables S13 to S21: Atomic coordinates of the optimized structures for the (C_4_H_6_)_2_Fe_2_(CO)_2_ complexes; Tables S22 to S27: Atomic coordinates of the optimized structures for the (C_4_H_6_)_2_Fe_2_(CO) complexes; Tables S28 to S36: Harmonic vibrational frequencies (in cm^−1^) and infrared intensities (in parentheses in km/mol) for the (C_4_H_6_)_2_Fe_2_(CO)_2_ complexes; Tables S37 to S42: Harmonic vibrational frequencies (in cm^−1^) and infrared intensities (in parentheses in km/mol) for the (C_4_H_6_)_2_Fe_2_(CO) complexes.
