# Supplementary material for: Highly Unsaturated Binuclear Butadiene Iron Carbonyls: Quintet Spin States, Perpendicular Structures, Agostic Hydrogen Atoms, and Iron-Iron Multiple Bonds
Source: Int J Mol Sci. 2011 Mar 30;12(4):2216–31. doi: 10.3390/ijms12042216 (PMC3127113; doi:10.3390/ijms12042216)
Supplement: Supplementary file 1 [file ijms-12-02216-s001.pdf]

Supporting Information

Tables S1 to S12: Fe–Fe distances (Å), total energies (E, in hartree), relative energies (*E*), zero-point energies (PE), enthalpies (*H*), free energies (*G*, in kcal/mol) and numbers of imaginary frequencies (Nimag) for $(C_4H_6)_2Fe_2(CO)_n$ (n = 2, 1) structures at M06-L, BP86 and B3LYP.

Tables S13 to S21: Atomic coordinates of the optimized structures for the $(C_4H_6)_2Fe_2(CO)_2$ complexes.

Tables S22 to S27: Atomic coordinates of the optimized structures for the $(C_4H_6)_2Fe_2(CO)_2$ complexes.

Tables S28 to S36: Harmonic vibrational frequencies (in cm^{-1}) and infrared intensities (in parentheses in km/mol) for the $(C_4H_6)_2Fe_2(CO)_2$ complexes.

Tables S37 to S42: Harmonic vibrational frequencies (in cm^{-1}) and infrared intensities (in parentheses in km/mol) for the mononuclear $C_4H_6Fe_2(CO)$ complexes.

Table S1. Fe–Fe distances (Å), total energies (*E*, in hartree), relative electronic energies (ΔE), zero-point energies (*ZPE*), enthalpies (ΔH), free energies (ΔG, in kcal/mol), numbers of imaginary frequencies (Nimag) and spin expectation values $\langle S^2 \rangle$ for the quintet $(C_4H_6)_2Fe_2(CO)_2$ structures at M06-L level.

		**2Q-1**(C_s)	**2Q-2**(C_1)	**2Q-3**(C_{2h})
M06-L	Fe–Fe	2.361	2.311	2.342
	E	−3066.14608	−3066.14371	−3066.13340
	ΔE	0.0	1.5	8.0
	ZPE	0.0	1.9	7.5
	ΔH	0.0	2.0	8.1
	ΔG	0.0	1.0	6.1
	Nimag	none	none	none
	$\langle S^2 \rangle$	6.30	6.31	6.63

Table S2. Fe–Fe distances (Å), total energies (*E*, in hartree), relative electronic energies (Δ*E*), zero-point energies (*ZPE*), enthalpies (Δ*H*), free energies (Δ*G*, in kcal/mol), numbers of imaginary frequencies (Nimag) and spin expectation values $\langle S^2 \rangle$ for the quintet $(C_4H_6)_2Fe_2(CO)_2$ structures at BP86 and B3LYP levels.

		**2Q-1**(C_2)	**2Q-2**(C_1)	**2Q-3**(C_2)
BP86	Fe–Fe	2.361	2.252	2.366
	E	−3066.72937	−3066.72243	−3066.71305
	Δ*E*	0.0	4.4	10.2
	ZPE	0.0	4.2	9.0
	Δ*H*	0.0	4.6	9.3
	Δ*G*	0.0	2.5	6.2
	Nimag	none	none	1(39i)
	$<S^2>$	6.10	6.09	6.20
B3LYP	Fe-Fe	2.408	2.395	2.446
	E	−3066.30315	−3066.31045	−3066.29342
	Δ*E*	0.0	−4.6	6.1
	ZPE	0.0	−4.7	5.1
	Δ*H*	0.0	−4.4	5.3
	Δ*G*	0.0	−6.2	3.9
	Nimag	none	none	1(2i)
	$<S^2>$	6.26	6.30	6.65

Table S3. Fe–Fe distances (Å), total energies (*E*, in hartree), relative electronic energies (Δ*E*), zero-point energies (*ZPE*), enthalpies (Δ*H*), free energies (Δ*G*, in kcal/mol), numbers of imaginary frequencies (Nimag) and spin expectation values $\langle S^2 \rangle$ for the triplet $(C_4H_6)_2Fe_2(CO)_2$ structures at M06-L level.

		**2T-1**(C_2)	**2T-2**(C_1)	**2T-3**(C_2)
M06-L	Fe–Fe	2.209	2.433	2.295
	E	−3066.13525	−3066.13271	−3066.1265
	Δ*E*	6.8	8.4	12.3
	ZPE	7.5	9.9	13.7
	Δ*H*	7.4	9.1	13.3
	Δ*G*	7.9	10.9	14.3
	Nimag	none	none	none
	$\langle S^2 \rangle$	2.20	2.12	2.21

Table S4. Fe–Fe distances (Å), total energies (*E*, in hartree), relative electronic energies (Δ*E*), zero-point energies (*ZPE*), enthalpies (Δ*H*), free energies (Δ*G*, in kcal/mol), numbers of imaginary frequencies (Nimag) and spin expectation values ⟨S^2⟩ for the triplet $(C_4H_6)_2Fe_2(CO)_2$ structures at BP86 and B3LYP levels.

		**2T-1**(C_2)	**2T-2**(C_1)	**2T-3**(C_2)
BP86	Fe–Fe	2.197	2.441	2.267
	E	−3066.73798	−3066.72155	−3066.73667
	Δ*E*	−5.4	4.9	−4.6
	ZPE	−5.4	5.2	−3.5
	Δ*H*	−5.3	4.7	−3.7
	Δ*G*	−5.3	5.5	−3.3
	Nimag	none	none	none
	$<S^2>$	2.06	2.05	2.07
B3LYP	Fe–Fe	2.211	2.539	2.381
	E	−3066.28605	−3066.28786	−3066.28491
	Δ*E*	10.7	9.6	11.4
	ZPE	11.3	10.2	12.5
	Δ*H*	11.2	9.6	12.3
	Δ*G*	11.3	10.6	12.6
	Nimag	none	none	none
	$<S^2>$	2.21	2.15	2.19

Table S5. Fe–Fe distances (Å), total energies (*E*, in hartree), relative electronic energies (Δ*E*), zero-point energies (*ZPE*), enthalpies (Δ*H*), free energies (Δ*G*, in kcal/mol) and numbers of imaginary frequencies (Nimag) for the singlet $(C_4H_6)_2Fe_2(CO)_2$ structures at M06-L level.

		**2S-1** (C_1)	**2S-2** (C_{2h})	**2S-3** (C_2)
M06-L	Fe–Fe	2.344	2.327	2.325
	E	−3066.11570	−3066.10797	−3066.09822
	Δ*E*	19.1	23.9	30.0
	ZPE	21.2	24.8	31.5
	Δ*H*	20.1	24.5	31.1
	Δ*G*	23.6	26.1	32.9
	Nimag	none	none	none

Table S6. Fe–Fe distances (Å), total energies (*E*, in hartree), relative energies (Δ*E*, in kcal/mol), relative electronic energies (Δ*E*), zero-point energies (*ZPE*), enthalpies (Δ*H*), free energies (Δ*G*, in kcal/mol) and numbers of imaginary frequencies (Nimag) for the singlet $(C_4H_6)_2Fe_2(CO)_2$ structures at BP86 and B3LYP levels.

		**2S-1** (C_1)	**2S-2** (C_{2h})	**2S-3** (C_2)
BP86	Fe-Fe	2.348	2.332	2.096
	E	−3066.71463	−3066.72813	−3066.71301
	Δ*E*	9.3	0.8	10.3
	ZPE	10.3	0.7	11.3
	Δ*H*	9.4	0.7	11.1
	Δ*G*	12.3	1.3	12.1
	Nimag	none	none	none
B3LYP	Fe–Fe	2.384	2.376	2.096
	E	−3066.25777	−3066.24558	−3066.24029
	Δ*E*	28.5	36.1	39.4
	ZPE	30.1	36.5	40.7
	Δ*H*	29.1	36.3	39.9
	Δ*G*	32.2	37.6	42.7
	Nimag	none	none	1(117i)

Table 7. Fe–Fe distances (Å), total energies (*E*, in hartree), relative electronic energies (Δ*E*), zero-point energies (*ZPE*), enthalpies (Δ*H*), free energies (Δ*G*, in kcal/mol), numbers of imaginary frequencies (Nimag) and spin expectation values $\langle S^2 \rangle$ for the quintet $(C_4H_6)_2Fe_2(CO)$ structures at M06-L level.

		**1Q-1** (C_1)	**1Q-2** (C_1)
M06-L	Fe–Fe	2.290	2.283
	E	−2952.77563	−2952.75505
	Δ*E*	0.0	12.9
	ZPE	0.0	12.3
	Δ*H*	0.0	12.6
	Δ*G*	0.0	11.7
	Nimag	none	none
	$\langle S^2 \rangle$	6.32	6.40

Table S8. Fe–Fe distances (Å), total energies (E, in hartree), relative electronic energies (ΔE), zero-point energies (ZPE), enthalpies (ΔH), free energies (ΔG, in kcal/mol), numbers of imaginary frequencies (Nimag) and spin expectation values $\langle S^2 \rangle$ for the quintet $(C_4H_6)_2Fe_2(CO)$ structures at BP86 and B3LYP levels.

		**1Q-1** (C_1)	**1Q-2** (C_1)
BP86	Fe–Fe	2.252	2.262
	E	−2953.34767	−2953.32122
	ΔE	0.0	16.6
	ZPE	0.0	15.8
	ΔH	0.0	16.1
	ΔG	0.0	15.0
	Nimag	none	none
	$<S^2>$	6.11	6.13
B3LYP	Fe–Fe	2.452	2.333
	E	−2952.92654	−2952.91399
	ΔE	0.0	7.9
	ZPE	0.0	7.2
	ΔH	0.0	7.4
	ΔG	0.0	8.1
	Nimag	none	none
	$<S^2>$	6.17	6.58

Table S9. Fe–Fe distances (Å), total energies (E, in hartree), relative electronic energies (ΔE), zero-point energies (ZPE), enthalpies (ΔH), free energies (ΔG, in kcal/mol), numbers of imaginary frequencies (Nimag) and spin expectation values $\langle S^2 \rangle$ for the triplet $(C_4H_6)_2Fe_2(CO)$ structures at M06-L method.

		**1T-1** (C_s)	**1T-2** (C_s)
M06-L	Fe–Fe	2.291	2.200
	E	−2952.75716	−2952.73705
	ΔE	11.6	24.2
	ZPE	11.1	24.3
	ΔH	11.3	24.1
	ΔG	10.1	24.9
	Nimag	none	none
	$\langle S^2 \rangle$	2.82	2.22

Table S10. Fe–Fe distances (Å), total energies (*E*, in hartree), relative electronic energies (Δ*E*), zero-point energies (*ZPE*), enthalpies (Δ*H*), free energies (Δ*G*, in kcal/mol), numbers of imaginary frequencies (Nimag) and spin expectation values ⟨S^2⟩ for the triplet $(C_4H_6)_2Fe_2(CO)$ structures at BP86 and B3LYP levels.

		**1T-1** (*Cs*)	**1T-2** (*Cs*)
BP86	Fe-Fe	2.183	2.108
	E	-2953.33791	-2953.32369
	Δ*E*	6.1	15.0
	ZPE	5.9	14.8
	Δ*H*	5.9	15.0
	Δ*G*	6.0	14.0
	Nimag	none	none
	<S^2>	2.27	2.08
B3LYP	Fe-Fe	2.652	2.366
	E	-2952.89218	-2952.86955
	Δ*E*	21.6	35.8
	ZPE	21.6	35.4
	Δ*H*	21.5	34.6
	Δ*G*	23.0	37.8
	Nimag	none	2(71i,50i)
	<S^2>	2.98	2.65

Table S11. Fe–Fe distances (Å), total energies (*E*, in hartree), relative electronic energies (Δ*E*), zero-point energies (*ZPE*), enthalpies (Δ*H*), free energies (Δ*G*, in kcal/mol) and numbers of imaginary frequencies (Nimag) for the singlet $(C_4H_6)_2Fe_2(CO)$ structures at M06-L method.

		**1S-1** (C_s)	**1S-2** (C_s)
M06-L	Fe–Fe	2.051	2.039
	E	−2952.70120	−2952.70002
	Δ*E*	46.7	47.4
	ZPE	46.8	47.7
	Δ*H*	46.5	47.8
	Δ*G*	47.9	48.3
	Nimag	none	none

Table S12. Fe–Fe distances (Å), total energies (*E*, in hartree), relative electronic energies (Δ*E*), zero-point energies (*ZPE*), enthalpies (Δ*H*), free energies (Δ*G*, in kcal/mol) and numbers of imaginary frequencies (Nimag) for the singlet $(C_4H_6)_2Fe_2(CO)$ structures at BP86 and B3LYP levels.

		**1S-1** (*Cs*)	**1S-2** (*Cs*)
BP86	Fe–Fe	2.051	2.041
	E	−2953.31292	−2953.30477
	Δ*E*	21.8	26.9
	ZPE	21.7	26.8
	Δ*H*	21.4	26.9
	Δ*G*	23.2	27.4
	Nimag	none	none
B3LYP	Fe–Fe	2.183	2.018
	E	−2952.84167	−2952.83950
	Δ*E*	53.3	54.6
	ZPE	54.0	55.4
	Δ*H*	55.1	53.6
	Δ*G*	58.2	56.6
	Nimag	none	none

Table S13. Optimized coordinates of the **2S-1** for the $(C_4H_6)_2Fe_2(CO)_2$ structure.

	BP86			M06-L			B3LYP		
	x	y	z	x	y	z	x	y	z
6	0.146085	-0.912287	1.152355	0.194413	-0.960504	1.123960	0.165894	-0.900835	1.161071
26	0.970274	0.146277	-0.177438	0.969586	0.158282	-0.169709	0.987098	0.160209	-0.186109
26	-1.316665	0.021927	0.339053	-1.307940	0.020931	0.367141	-1.326757	0.011864	0.367928
6	1.836811	-1.064240	-1.564235	1.824245	-1.025410	-1.571367	1.917073	-1.071180	-1.542769
6	-0.387504	1.759696	1.133925	-0.369107	1.731211	1.111894	-0.519062	1.812232	1.108149
6	2.441458	-1.240902	-0.270654	2.433193	-1.211024	-0.298515	2.515314	-1.191772	-0.256092
6	-1.630206	2.069615	0.451282	-1.634999	2.032141	0.483078	-1.742138	2.045071	0.373045
6	2.903803	-0.062443	0.411408	2.883025	-0.047631	0.392538	2.929354	-0.005427	0.418452
6	-1.771066	1.639646	-0.896045	-1.802601	1.605731	-0.847291	-1.825783	1.560142	-0.946768
6	2.752372	1.182705	-0.290282	2.720240	1.187392	-0.300961	2.745419	1.234007	-0.261946
6	-0.680192	0.799436	-1.400620	-0.703297	0.818761	-1.380089	-0.696333	0.766770	-1.415133
6	-2.267739	-1.281208	-0.354014	-2.281951	-1.271515	-0.356425	-2.261748	-1.347300	-0.333628
8	-2.912284	-2.166639	-0.797967	-2.919745	-2.123953	-0.834194	-2.862186	-2.230126	-0.789308
1	2.324567	-0.447789	-2.333632	2.288900	-0.382518	-2.320348	2.356441	-0.430356	-2.307144
1	1.247551	-1.902110	-1.962521	1.261768	-1.861368	-1.985158	1.395196	-1.944282	-1.934194
1	2.363826	-2.186839	0.280974	2.355526	-2.152463	0.241748	2.456834	-2.121459	0.308153
1	3.175614	-0.116125	1.473823	3.159548	-0.111052	1.443123	3.182449	-0.053408	1.476838
1	3.057493	1.267301	-1.343250	3.023462	1.269557	-1.346225	3.035449	1.339030	-1.307726
1	2.887724	2.109742	0.286806	2.856533	2.107359	0.268888	2.858118	2.148743	0.322411
8	0.328607	-1.614404	2.113773	0.394887	-1.685317	2.047489	0.387269	-1.591876	2.101833
1	-2.483779	2.508009	0.987597	-2.465765	2.463290	1.039656	-2.627203	2.458409	0.858472
1	0.571920	2.055685	0.653816	0.546205	2.077713	0.605087	0.428671	2.142290	0.663578
1	-0.354268	1.859983	2.226608	-0.299624	1.851586	2.191330	-0.541410	1.973722	2.186111
1	-2.695340	1.805077	-1.462679	-2.738133	1.745284	-1.382117	-2.745370	1.650001	-1.522026
1	0.371712	1.297402	-1.420386	0.305785	1.347004	-1.412695	0.309652	1.301797	-1.448799
1	-0.854349	0.284607	-2.354318	-0.877121	0.305316	-2.323803	-0.846281	0.227380	-2.349442

Table S14. Optimized coordinates of the **2S-2** for the $(C_4H_6)_2Fe_2(CO)_2$ structure.

	BP86			M06-L			B3LYP		
	x	y	z	x	y	z	x	y	z
6	1.427083	−0.187073	0.000000	1.429430	−0.211861	0.000000	1.402234	−0.246996	0.000000
26	0.070913	1.164052	0.000000	0.069164	1.161336	0.000000	0.095277	1.184294	0.000000
26	−0.070913	−1.164052	0.000000	−0.069164	−1.161336	0.000000	−0.095277	−1.184294	0.000000
6	0.514597	2.443934	1.511494	0.515673	2.434300	1.488879	0.509576	2.496928	1.509088
6	−0.514597	−2.443934	1.511494	−0.515673	−2.434300	1.488879	−0.509576	−2.496928	1.509088
6	−0.514597	3.046036	0.718342	−0.515673	3.018690	0.711688	−0.509576	3.072882	0.715195
6	0.514597	−3.046036	0.718342	0.515673	−3.018690	0.711688	0.509576	−3.072882	0.715195
6	−0.514597	3.046036	−0.718342	−0.515673	3.018690	−0.711688	−0.509576	3.072882	−0.715195
6	0.514597	−3.046036	−0.718342	0.515673	−3.018690	−0.711688	0.509576	−3.072882	−0.715195
6	0.514597	2.443934	−1.511494	0.515673	2.434300	−1.488879	0.509576	2.496928	−1.509088
6	−0.514597	−2.443934	−1.511494	−0.515673	−2.434300	−1.488879	−0.509576	−2.496928	−1.509088
6	−1.427083	0.187073	0.000000	−1.429430	0.211861	0.000000	−1.402234	0.246996	0.000000
8	−2.634840	0.128687	0.000000	−2.619686	0.088070	0.000000	−2.582094	0.056407	0.000000
1	1.581523	2.524093	1.239262	1.566634	2.511100	1.188853	1.565600	2.556190	1.225338
1	0.332166	2.305753	2.582914	0.364884	2.314425	2.556556	0.335107	2.377462	2.575234
1	−1.487971	3.227156	1.195249	−1.480455	3.185872	1.187447	−1.475884	3.254561	1.186390
1	−1.487971	3.227156	−1.195249	−1.480455	3.185872	−1.187447	−1.475884	3.254561	−1.186390
1	1.581523	2.524093	−1.239262	1.566634	2.511100	−1.188853	1.565600	2.556190	−1.225338
1	0.332166	2.305753	−2.582914	0.364884	2.314425	−2.556556	0.335107	2.377462	−2.575234
8	2.634840	−0.128687	0.000000	2.619686	−0.088070	0.000000	2.582094	−0.056407	0.000000
1	1.487971	−3.227156	1.195249	1.480455	−3.185872	1.187447	1.475884	−3.254561	1.186390
1	−1.581523	−2.524093	1.239262	−1.566634	−2.511100	1.188853	−1.565600	−2.556190	1.225338
1	−0.332166	−2.305753	2.582914	−0.364884	−2.314425	2.556556	−0.335107	−2.377462	2.575234
1	1.487971	−3.227156	−1.195249	1.480455	−3.185872	−1.187447	1.475884	−3.254561	−1.186390
1	−1.581523	−2.524093	−1.239262	−1.566634	−2.511100	−1.188853	−1.565600	−2.556190	−1.225338
1	−0.332166	−2.305753	−2.582914	−0.364884	−2.314425	−2.556556	−0.335107	−2.377462	−2.575234

Table S15. Optimized coordinates of the **2S-3** for the $(C_4H_6)_2Fe_2(CO)_2$ structure.

	BP86			M06-L			B3LYP		
	x	y	z	x	y	z	x	y	z
6	−0.464340	1.633969	1.375043	−0.539491	1.522887	1.397434	−0.463870	1.979480	1.366955
26	−0.183640	1.154028	−0.259835	−0.211170	1.143210	−0.261881	−0.088259	1.044431	−0.067233
26	0.183640	−1.154028	−0.259835	0.211170	−1.143210	−0.261881	0.088259	−1.044431	−0.067233
6	1.735872	1.954375	0.057524	1.689438	1.937559	0.098655	1.779576	1.950569	0.045297
6	−2.209303	1.067163	−0.420614	−2.214180	0.994350	−0.493877	−2.057430	1.083444	−0.726490
6	1.636904	1.310775	−1.204138	1.592801	1.363258	−1.181796	1.527869	1.410639	−1.236672
6	−1.735872	0.129649	−1.395082	−1.689438	0.055642	−1.422510	−1.527869	0.021662	−1.509061
6	1.735872	−0.129649	−1.395082	1.689438	−0.055642	−1.422510	1.527869	−0.021662	−1.509061
6	−1.636904	−1.310775	−1.204138	−1.592801	−1.363258	−1.181796	−1.527869	−1.410639	−1.236672
6	2.209303	−1.067163	−0.420614	2.214180	−0.994350	−0.493877	2.057430	−1.083444	−0.726490
6	−1.735872	−1.954375	0.057524	−1.689438	−1.937559	0.098655	−1.779576	−1.950569	0.045297
6	0.464340	−1.633969	1.375043	0.539491	−1.522887	1.397434	0.463870	−1.979480	1.366955
8	0.639480	−1.886564	2.517609	0.734702	−1.672689	2.541262	0.680201	−2.551089	2.359364
1	2.220929	1.462776	0.909107	2.161910	1.397675	0.917284	2.341347	1.387967	0.790709
1	1.751148	3.051941	0.086869	1.712970	3.020837	0.190847	1.824311	3.031041	0.159403
1	1.457491	1.923258	−2.103795	1.386752	2.006557	−2.040918	1.312054	2.073137	−2.074723
1	1.591028	−0.479525	−2.429445	1.512139	−0.376331	−2.450581	1.196308	−0.287423	−2.513306
1	2.769404	−0.697734	0.447585	2.799412	−0.625881	0.346809	2.733192	−0.864585	0.099188
1	2.529666	−2.062041	−0.771875	2.540669	−1.965924	−0.872069	2.239112	−2.032895	−1.228304
8	−0.639480	1.886564	2.517609	−0.734702	1.672689	2.541262	−0.680201	2.551089	2.359364
1	−1.591028	0.479525	−2.429445	−1.512139	0.376331	−2.450581	−1.196308	0.287423	−2.513306
1	−2.769404	0.697734	0.447585	−2.799412	0.625881	0.346809	−2.733192	0.864585	0.099188
1	−2.529666	2.062041	−0.771875	−2.540669	1.965924	−0.872069	−2.239112	2.032895	−1.228304
1	−1.457491	−1.923258	−2.103795	−1.386752	−2.006557	−2.040918	−1.312054	−2.073137	−2.074723
1	−2.220929	−1.462776	0.909107	−2.161910	−1.397675	0.917284	−2.341347	−1.387967	0.790709
1	−1.751148	−3.051941	0.086869	−1.712970	−3.020837	0.190847	−1.824311	−3.031041	0.159403

Table S16. Optimized coordinates of the **2T-1** for the **(C_4H_6)**$_2$$Fe_2(CO)_2$ structure.

	BP86			M06-L			B3LYP		
	x	y	z	x	y	z	x	y	z
6	−1.532530	0.232732	−0.333106	−1.566563	0.271587	−0.309605	−1.575827	0.276692	−0.307316
26	0.054510	1.097000	0.063052	0.043402	1.103555	0.031913	0.044753	1.104753	0.031938
26	−0.054510	−1.097000	0.063052	−0.043402	−1.103555	0.031913	−0.044753	−1.104753	0.031938
6	−0.054510	2.657874	−1.307364	−0.043402	2.703265	−1.266571	−0.044753	2.737327	−1.283904
6	0.054510	−2.657874	−1.307364	0.043402	−2.703265	−1.266571	0.044753	−2.737327	−1.283904
6	1.117286	2.798083	−0.498570	1.155584	2.751905	−0.510631	1.138786	2.805366	−0.503762
6	−1.117286	−2.798083	−0.498570	−1.155584	−2.751905	−0.510631	−1.138786	−2.805366	−0.503762
6	1.047303	2.686508	0.934437	1.130325	2.595418	0.905806	1.103724	2.650831	0.915870
6	−1.047303	−2.686508	0.934437	−1.130325	−2.595418	0.905806	−1.103724	−2.650831	0.915870
6	−0.192672	2.438126	1.610926	−0.094678	2.396843	1.602904	−0.117784	2.435013	1.614438
6	0.192672	−2.438126	1.610926	0.094678	−2.396843	1.602904	0.117784	−2.435013	1.614438
6	1.532530	−0.232732	−0.333106	1.566563	−0.271587	−0.309605	1.575827	−0.276692	−0.307316
8	2.683560	−0.176363	−0.644723	2.714089	−0.158328	−0.559257	2.718069	−0.215556	−0.570210
1	−1.010929	3.116989	−1.019134	−0.950927	3.193009	−0.911630	−0.971317	3.205977	−0.952121
1	0.072679	2.506947	−2.385871	0.024014	2.609572	−2.346327	0.051271	2.637055	−2.362842
1	2.109240	2.716196	−0.961836	2.114101	2.624932	−1.009609	2.108370	2.711106	−0.991814
1	1.993398	2.523579	1.469115	2.072981	2.358053	1.396254	2.050063	2.452708	1.419136
1	−1.122062	2.944762	1.312623	−0.986824	2.968499	1.343231	−1.025275	2.980263	1.352854
1	−0.154493	2.141915	2.665599	−0.051288	2.081312	2.640987	−0.064922	2.120296	2.654133
8	−2.683560	0.176363	−0.644723	−2.714089	0.158328	−0.559257	−2.718069	0.215556	−0.570210
1	−2.109240	−2.716196	−0.961836	−2.114101	−2.624932	−1.009609	−2.108370	−2.711106	−0.991814
1	1.010929	−3.116989	−1.019134	0.950927	−3.193009	−0.911630	0.971317	−3.205977	−0.952121
1	−0.072679	−2.506947	−2.385871	−0.024014	−2.609572	−2.346327	−0.051271	−2.637055	−2.362842
1	−1.993398	−2.523579	1.469115	−2.072981	−2.358053	1.396254	−2.050063	−2.452708	1.419136
1	1.122062	−2.944762	1.312623	0.986824	−2.968499	1.343231	1.025275	−2.980263	1.352854
1	0.154493	−2.141915	2.665599	0.051288	−2.081312	2.640987	0.064922	−2.120296	2.654133

Table S17. Optimized coordinates of *the* **2T-2** for the $(C_4H_6)_2Fe_2(CO)_2$ structure.

	BP86			M06-L			B3LYP		
	x	y	z	x	y	z	x	y	z
6	−0.599237	1.601457	−0.503953	−0.550413	1.582553	−0.531461	−0.674801	1.640143	−0.675361
26	1.157987	−0.256783	0.133700	1.153486	−0.229479	0.111173	1.189142	−0.310341	0.160978
26	−1.238460	−0.030082	−0.270483	−1.238516	−0.037406	−0.292165	−1.284518	0.015385	−0.311135
6	2.068824	1.038684	1.438105	2.042226	1.072111	1.393615	2.106712	0.783159	1.671248
6	−0.087153	−1.480965	−1.259671	−0.123257	−1.543275	−1.232867	−0.127697	−1.373401	−1.403167
6	2.381776	1.369843	0.074664	2.411929	1.335722	0.046425	2.354041	1.364945	0.400250
6	−1.254639	−2.071664	−0.626612	−1.305257	−2.066534	−0.591200	−1.278453	−2.010667	−0.795263
6	2.889544	0.333811	−0.780405	2.914047	0.255877	−0.739701	2.872243	0.554487	−0.655448
6	−1.404138	−1.811979	0.772757	−1.443916	−1.774958	0.788481	−1.405814	−1.871231	0.602371
6	3.084949	−0.959053	−0.186337	3.038678	−1.001830	−0.088581	3.148375	−0.812766	−0.383763
6	−0.334543	−1.049207	1.412751	−0.358405	−1.047403	1.414128	−0.299267	−1.235764	1.321984
6	−2.850837	0.596022	0.138647	−2.830749	0.648561	0.142549	−2.828502	0.627578	0.363905
8	−3.917733	1.025580	0.404175	−3.864604	1.108612	0.431154	−3.827160	1.027792	0.806941
1	2.763209	0.444148	2.049241	2.685319	0.478416	2.044273	2.811273	0.081761	2.118274
1	1.460896	1.753611	2.007131	1.443687	1.820730	1.908711	1.513113	1.349881	2.385735
1	2.046356	2.315360	−0.369411	2.104334	2.254805	−0.447823	1.952051	2.346424	0.154587
1	2.945985	0.494923	−1.865505	3.004197	0.362886	−1.818925	2.857929	0.937748	−1.675479
1	3.561474	−1.056461	0.799364	3.463414	−1.064452	0.913742	3.634378	−1.115144	0.543546
1	3.261922	−1.814061	−0.853474	3.212332	−1.885602	−0.700682	3.342385	−1.474074	−1.227810
8	−0.339544	2.750649	−0.632617	−0.250438	2.712231	−0.640302	−0.348605	2.734485	−0.917325
1	−2.051030	−2.564681	−1.197577	−2.110303	−2.542159	−1.145089	−2.086975	−2.436067	−1.388223
1	0.915902	−1.794268	−0.802748	0.844727	−1.868224	−0.767706	0.869217	−1.723919	−1.024094
1	−0.002750	−1.535361	−2.353449	−0.044669	−1.635679	−2.314369	−0.082226	−1.329875	−2.491307
1	−2.312354	−2.109829	1.309971	−2.357132	−2.023665	1.322164	−2.305164	−2.211651	1.112716
1	0.693609	−1.561134	1.333115	0.630336	−1.584933	1.342129	0.654810	−1.829163	1.223291
1	−0.499970	−0.725274	2.447557	−0.514406	−0.704798	2.434236	−0.475896	−1.024172	2.375367

Table S18. Optimized *coordinates* of the **2T-3** for the $(C_4H_6)_2Fe_2(CO)_2$ structure.

	BP86			M06-L			B3LYP		
	x	y	z	x	y	z	x	y	z
6	−0.477645	1.830691	1.351326	−0.572487	1.769241	1.378280	−0.594674	1.885707	1.359460
26	−0.191090	1.117079	−0.208547	−0.211025	1.128074	−0.206321	−0.191127	1.174822	−0.188373
26	0.191090	−1.117079	−0.208547	0.211025	−1.128074	−0.206321	0.191127	−1.174822	−0.188373
6	1.690745	2.051263	0.021778	1.592560	2.192761	0.020260	1.644351	2.261850	−0.014960
6	−2.213921	0.983799	−0.441294	−2.181985	0.773768	−0.480524	−2.175609	0.775894	−0.441311
6	1.561273	1.388095	−1.228328	1.470030	1.567369	−1.234044	1.520584	1.581354	−1.239379
6	−1.690745	0.055295	−1.401126	−1.592560	−0.137680	−1.408724	−1.644351	−0.149027	−1.394127
6	1.690745	−0.055295	−1.401126	1.592560	0.137680	−1.408724	1.644351	0.149027	−1.394127
6	−1.561273	−1.388095	−1.228328	−1.470030	−1.567369	−1.234044	−1.520584	−1.581354	−1.239379
6	2.213921	−0.983799	−0.441294	2.181985	−0.773768	−0.480524	2.175609	−0.775894	−0.441311
6	−1.690745	−2.051263	0.021778	−1.592560	−2.192761	0.020260	−1.644351	−2.261850	−0.014960
6	0.477645	−1.830691	1.351326	0.572487	−1.769241	1.378280	0.594674	−1.885707	1.359460
8	0.636678	−2.227008	2.454608	0.763401	−2.116353	2.480099	0.834856	−2.291307	2.427264
1	2.214146	1.579030	0.862205	2.138856	1.712756	0.830686	2.176868	1.822222	0.826318
1	1.674787	3.147129	0.039652	1.537256	3.275249	0.079122	1.581737	3.347101	−0.008769
1	1.339687	1.981155	−2.129407	1.197198	2.160236	−2.108019	1.259604	2.152307	−2.133227
1	1.529206	−0.419618	−2.427585	1.407576	−0.223582	−2.421819	1.478942	−0.218855	−2.408409
1	2.760470	−0.610577	0.433990	2.738828	−0.376247	0.366136	2.726221	−0.397225	0.417616
1	2.557858	−1.962436	−0.807633	2.578558	−1.711633	−0.874026	2.562338	−1.721590	−0.832048
8	−0.636678	2.227008	2.454608	−0.763401	2.116353	2.480099	−0.834856	2.291307	2.427264
1	−1.529206	0.419618	−2.427585	−1.407576	0.223582	−2.421819	−1.478942	0.218855	−2.408409
1	−2.760470	0.610577	0.433990	−2.738828	0.376247	0.366136	−2.726221	0.397225	0.417616
1	−2.557858	1.962436	−0.807633	−2.578558	1.711633	−0.874026	−2.562338	1.721590	−0.832048
1	−1.339687	−1.981155	−2.129407	−1.197198	−2.160236	−2.108019	−1.259604	−2.152307	−2.133227
1	−2.214146	−1.579030	0.862205	−2.138856	−1.712756	0.830686	−2.176868	−1.822222	0.826318
1	−1.674787	−3.147129	0.039652	−1.537256	−3.275249	0.079122	−1.581737	−3.347101	−0.008769

Table S19. Optimized coordinates *of* the **2Q-1** for the $(C_4H_6)_2Fe_2(CO)_2$ structure.

	BP86			M06-L			B3LYP		
	x	y	z	x	y	z	x	y	z
6	0.927514	2.443709	0.000000	0.969542	2.480912	0.000000	1.093909	2.450950	0.000000
26	0.751554	0.656958	0.000000	0.730559	0.676868	0.000000	0.714777	0.676932	0.000000
26	−0.875025	−1.054748	0.000000	−0.858740	−1.068579	0.000000	−0.905538	−1.104784	0.000000
6	1.111990	0.571809	2.054017	1.089764	0.532124	2.049515	1.072044	0.559213	2.080910
6	1.111990	0.571809	−2.054017	1.089764	0.532124	−2.049515	1.072044	0.559213	−2.080910
6	1.111990	−0.789820	1.612206	1.089764	−0.806487	1.578430	1.072044	−0.787341	1.638643
6	1.111990	−0.789820	−1.612206	1.089764	−0.806487	−1.578430	1.072044	−0.787341	−1.638643
6	0.031671	−1.781772	1.727004	0.029650	−1.799278	1.709340	0.000948	−1.781017	1.759861
6	0.031671	−1.781772	−1.727004	0.029650	−1.799278	−1.709340	0.000948	−1.781017	−1.759861
6	−1.325405	−1.505611	2.031111	−1.315555	−1.516781	2.015950	−1.348572	−1.541793	2.071954
6	−1.325405	−1.505611	−2.031111	−1.315555	−1.516781	−2.015950	−1.348572	−1.541793	−2.071954
6	−1.157674	0.904778	0.000000	−1.141074	0.998297	0.000000	−1.107321	1.078828	0.000000
8	−2.081786	1.662971	0.000000	−2.104877	1.679195	0.000000	−2.081061	1.737363	0.000000
1	0.270335	0.960643	2.640732	0.253868	0.893467	2.646443	0.234913	0.944706	2.659476
1	2.080596	1.033566	2.286878	2.047524	0.988048	2.290702	2.031856	1.017616	2.313738
1	2.090974	−1.228324	1.362420	2.057377	−1.228885	1.300554	2.042600	−1.221797	1.389507
1	0.328923	−2.830645	1.570511	0.324153	−2.837847	1.552017	0.312004	−2.818056	1.610864
1	−1.625658	−0.554408	2.489194	−1.593789	−0.561200	2.460771	−1.673752	−0.590268	2.490710
1	−2.007627	−2.348969	2.195485	−1.991685	−2.342654	2.218030	−2.002275	−2.390989	2.258656
8	0.975930	3.618609	0.000000	1.035320	3.642360	0.000000	1.280062	3.596925	0.000000
1	2.090974	−1.228324	−1.362420	2.057377	−1.228885	−1.300554	2.042600	−1.221797	−1.389507
1	0.270335	0.960643	−2.640732	0.253868	0.893467	−2.646443	0.234913	0.944706	−2.659476
1	2.080596	1.033566	−2.286878	2.047524	0.988048	−2.290702	2.031856	1.017616	−2.313738
1	0.328923	−2.830645	−1.570511	0.324153	−2.837847	−1.552017	0.312004	−2.818056	−1.610864
1	−1.625658	−0.554408	−2.489194	−1.593789	−0.561200	−2.460771	−1.673752	−0.590268	−2.490710
1	−2.007627	−2.348969	−2.195485	−1.991685	−2.342654	−2.218030	−2.002275	−2.390989	−2.258656

Table S20. Optimized coordinates of the **2Q-2** for the $(C_4H_6)_2Fe_2(CO)_2$ structure.

	BP86			M06-L			B3LYP		
	x	y	z	x	y	z	x	y	z
6	−0.509992	1.765465	0.716504	0.187015	−1.144790	1.352222	0.297025	−0.333727	1.774708
26	0.910209	−0.249450	−0.399944	−0.910989	−0.043881	−0.501912	−0.997804	−0.077277	−0.415118
26	−1.189280	0.194466	0.283175	1.225541	−0.048471	0.379836	1.272609	0.051928	0.336306
6	2.537790	−1.624648	0.144594	−2.366172	1.559781	−0.764089	−2.572007	1.307558	−1.076202
6	−2.443336	0.942970	−1.244308	2.445376	−1.549165	−0.446271	2.255194	−1.838538	0.176052
6	2.870953	−0.344067	0.634252	−2.770778	0.756968	0.305367	−2.977502	0.765931	0.149608
6	−2.816164	−0.392132	−0.892801	2.971424	−0.262770	−0.727719	2.846355	−0.879386	−0.689112
6	2.780748	0.884037	−0.119886	−2.832073	−0.675816	0.264989	−2.975577	−0.633536	0.488278
6	−1.837880	−1.430179	−0.905455	2.119801	0.705758	−1.309397	2.037781	−0.133760	−1.583551
6	2.331047	0.978975	−1.458423	−2.517609	−1.441460	−0.864384	−2.552128	−1.667100	−0.349761
6	−0.514192	−1.278084	−1.555142	0.791406	0.330045	−1.806614	0.627388	−0.480500	−1.826114
6	−0.875229	−0.713768	1.779118	0.869643	1.524972	1.173091	1.282117	1.857547	0.394016
8	−0.693236	−1.277642	2.802006	0.600114	2.536626	1.695003	1.313570	3.020130	0.457707
1	2.674561	−1.888924	−0.915748	−2.537102	1.251176	−1.798177	−2.672647	0.746660	−2.009269
1	2.489690	−2.470534	0.838627	−2.211357	2.623028	−0.614242	−2.501349	2.386109	−1.188855
1	2.966058	−0.227210	1.723378	−2.787349	1.207115	1.297504	−3.097906	1.452311	0.988931
1	2.815110	1.810992	0.469248	−2.911565	−1.184070	1.224054	−3.097203	−0.862256	1.547487
1	2.527382	0.177995	−2.190321	−2.704369	−1.061129	−1.872772	−2.625357	−1.593150	−1.438309
1	2.152747	1.970198	−1.889448	−2.478181	−2.522410	−0.776424	−2.471292	−2.677356	0.042708
8	−0.082199	2.826791	1.018822	−0.379279	−1.888352	2.066818	−0.266196	−0.589793	2.769350
1	−3.763100	−0.578709	−0.367373	3.912374	0.064301	−0.288744	3.861028	−0.527029	−0.500533
1	−1.744564	1.124424	−2.071496	1.785932	−2.033319	−1.165399	1.465379	−2.495549	−0.182477
1	−3.151521	1.757138	−1.051124	3.026294	−2.231798	0.167476	2.856579	−2.246012	0.986245
1	−2.104360	−2.402279	−0.470371	2.462548	1.733586	−1.399054	2.488715	0.694579	−2.128096
1	−0.536290	−0.716924	−2.508521	0.784396	−0.599818	−2.393266	0.449050	−1.552497	−1.979245
1	−0.008866	−2.251189	−1.688194	0.325168	1.147138	−2.364734	0.209192	0.103612	−2.653477

Table S21. Optimized coordinates of the **2Q-3** *for* the $(C_4H_6)_2Fe_2(CO)_2$ structure.

	BP86			M06-L			B3LYP		
	x	y	z	x	y	z	x	y	z
6	1.589380	0.134182	0.000000	1.632640	0.031623	0.000000	−0.609296	1.567446	0.000000
26	0.116867	1.177401	0.000000	0.190186	1.155555	0.000000	−1.216701	−0.125259	0.000000
26	−0.116867	−1.177401	0.000000	−0.190186	−1.155555	0.000000	1.216701	0.125259	0.000000
6	0.556980	2.640843	1.510206	0.558393	2.654742	1.497218	−2.814025	−0.168544	1.508392
6	−0.556980	−2.640843	1.510206	−0.558393	−2.654742	1.497218	2.814025	0.168544	1.508392
6	−0.556980	3.045683	0.722736	−0.558393	3.004002	0.716435	−2.814025	−1.336862	0.719214
6	0.556980	−3.045683	0.722736	0.558393	−3.004002	0.716435	2.814025	1.336862	0.719214
6	−0.556980	3.045683	−0.722736	−0.558393	3.004002	−0.716435	−2.814025	−1.336862	−0.719214
6	0.556980	−3.045683	−0.722736	0.558393	−3.004002	−0.716435	2.814025	1.336862	−0.719214
6	0.556980	2.640843	−1.510206	0.558393	2.654742	−1.497218	−2.814025	−0.168544	−1.508392
6	−0.556980	−2.640843	−1.510206	−0.558393	−2.654742	−1.497218	2.814025	0.168544	−1.508392
6	−1.589380	−0.134182	0.000000	−1.632640	−0.031623	0.000000	0.609296	−1.567446	0.000000
8	−2.726662	0.230462	0.000000	−2.721248	0.430903	0.000000	0.505465	−2.739154	0.000000
1	1.588746	2.864950	1.205663	1.571149	2.880564	1.161364	−3.328822	0.737434	1.190914
1	0.415345	2.496489	2.587117	0.444807	2.546674	2.570381	−2.657016	−0.258664	2.580325
1	−1.540722	3.120665	1.207188	−1.536237	3.033390	1.194634	−2.579641	−2.286897	1.200629
1	−1.540722	3.120665	−1.207188	−1.536237	3.033390	−1.194634	−2.579641	−2.286897	−1.200629
1	1.588746	2.864950	−1.205663	1.571149	2.880564	−1.161364	−3.328822	0.737434	−1.190914
1	0.415345	2.496489	−2.587117	0.444807	2.546674	−2.570381	−2.657016	−0.258664	−2.580325
8	2.726662	−0.230462	0.000000	2.721248	−0.430903	0.000000	−0.505465	2.739154	0.000000
1	1.540722	−3.120665	1.207188	1.536237	−3.033390	1.194634	2.579641	2.286897	1.200629
1	−1.588746	−2.864950	1.205663	−1.571149	−2.880564	1.161364	3.328822	−0.737434	1.190914
1	−0.415345	−2.496489	2.587117	−0.444807	−2.546674	2.570381	2.657016	0.258664	2.580325
1	1.540722	−3.120665	−1.207188	1.536237	−3.033390	−1.194634	2.579641	2.286897	−1.200629
1	−1.588746	−2.864950	−1.205663	−1.571149	−2.880564	−1.161364	3.328822	−0.737434	−1.190914
1	−0.415345	−2.496489	−2.587117	−0.444807	−2.546674	−2.570381	2.657016	0.258664	−2.580325

Table S22. Optimized coordinates of *the* **1S-1** for the $(C_4H_6)_2Fe_2(CO)$ structure.

	BP86			M06-L			B3LYP		
	x	y	z	x	y	z	x	y	z
26	0.060254	−1.136619	0.000000	0.086626	−1.131791	0.000000	0.297122	−1.194098	0.000000
26	0.148943	0.912043	0.000000	0.137737	0.918822	0.000000	−0.014046	0.966581	0.000000
6	−0.123607	−1.675925	1.886907	−0.145954	−1.651615	1.878517	−0.203415	−1.791936	1.851133
6	−0.123607	−1.675925	−1.886907	−0.145954	−1.651615	−1.878517	−0.203415	−1.791936	−1.851133
6	−1.283933	−0.896718	1.498596	−1.278971	−0.882162	1.455182	−1.237796	−0.900855	1.485341
6	−1.283933	−0.896718	−1.498596	−1.278971	−0.882162	−1.455182	−1.237796	−0.900855	−1.485341
6	−1.283933	0.582607	1.531984	−1.278971	0.585366	1.494311	−1.237796	0.552351	1.616288
6	−1.283933	0.582607	−1.531984	−1.278971	0.585366	−1.494311	−1.237796	0.552351	−1.616288
6	−0.149352	1.345443	1.966603	−0.153912	1.321705	1.959091	−0.090939	1.295147	2.023958
6	−0.149352	1.345443	−1.966603	−0.153912	1.321705	−1.959091	−0.090939	1.295147	−2.023958
6	1.908727	1.040966	0.000000	1.907284	1.021422	0.000000	1.716858	1.236122	0.000000
8	3.085173	1.161130	0.000000	3.074160	1.107822	0.000000	2.870771	1.430901	0.000000
1	0.668615	−1.255890	2.525180	0.626830	−1.216585	2.516495	0.626806	−1.441989	2.471571
1	−0.257776	−2.764521	1.987184	−0.278702	−2.727095	2.000574	−0.419397	−2.858581	1.922060
1	−2.250795	−1.397619	1.349479	−2.230955	−1.378559	1.269916	−2.181646	−1.342054	1.163174
1	−2.237505	1.093992	1.343648	−2.220998	1.096004	1.302383	−2.198676	1.054953	1.499083
1	0.617010	0.911802	2.621205	0.588509	0.864218	2.611374	0.702194	0.828300	2.604775
1	−0.281028	2.431860	2.087658	−0.273045	2.395282	2.109365	−0.223249	2.356875	2.239919
1	−2.250795	−1.397619	−1.349479	−2.230955	−1.378559	−1.269916	−2.181646	−1.342054	−1.163174
1	0.668615	−1.255890	−2.525180	0.626830	−1.216585	−2.516495	0.626806	−1.441989	−2.471571
1	−0.257776	−2.764521	−1.987184	−0.278702	−2.727095	−2.000574	−0.419397	−2.858581	−1.922060
1	−2.237505	1.093992	−1.343648	−2.220998	1.096004	−1.302383	−2.198676	1.054953	−1.499083
1	0.617010	0.911802	−2.621205	0.588509	0.864218	−2.611374	0.702194	0.828300	−2.604775
1	−0.281028	2.431860	−2.087658	−0.273045	2.395282	−2.109365	−0.223249	2.356875	−2.239919

Table S23. Optimized coordinates of the **1S-2** for the $(C_4H_6)_2Fe_2(CO)$ structure.

	BP86			M06-L			B3LYP		
	x	y	z	x	y	z	x	y	z
6	1.736214	0.264574	0.000000	1.768010	0.297832	0.000000	1.782582	0.270266	0.000000
26	0.361602	−1.041742	0.000000	0.400677	−1.045188	0.000000	0.389662	−1.026983	0.000000
26	−0.012587	0.964780	0.000000	0.010952	0.956365	0.000000	0.027219	0.958386	0.000000
6	−0.769549	−1.777509	1.537016	−0.780802	−1.705110	1.513699	−0.786200	−1.757164	1.547499
6	−0.309900	2.264334	1.516084	−0.355279	2.231138	1.498200	−0.364056	2.253287	1.513048
6	−0.309900	−2.865188	0.722127	−0.355279	−2.800371	0.715813	−0.364056	−2.825155	0.717943
6	−1.505406	2.180302	0.721146	−1.532195	2.064858	0.714455	−1.533786	2.111172	0.714915
6	−0.309900	−2.865188	−0.722127	−0.355279	−2.800371	−0.715813	−0.364056	−2.825155	−0.717943
6	−1.505406	2.180302	−0.721146	−1.532195	2.064858	−0.714455	−1.533786	2.111172	−0.714915
6	−0.769549	−1.777509	−1.537016	−0.780802	−1.705110	−1.513699	−0.786200	−1.757164	−1.547499
6	−0.309900	2.264334	−1.516084	−0.355279	2.231138	−1.498200	−0.364056	2.253287	−1.513048
1	−1.721949	−1.262567	1.330980	−1.701543	−1.161238	1.285122	−1.690310	−1.184422	1.337396
1	−0.492999	−1.799064	2.598578	−0.532862	−1.730169	2.571902	−0.516210	−1.806419	2.600561
1	0.379780	−3.586320	1.186474	0.298853	−3.538723	1.179945	0.300508	−3.564929	1.169136
1	0.379780	−3.586320	−1.186474	0.298853	−3.538723	−1.179945	0.300508	−3.564929	−1.169136
1	−1.721949	−1.262567	−1.330980	−1.701543	−1.161238	−1.285122	−1.690310	−1.184422	−1.337396
1	−0.492999	−1.799064	−2.598578	−0.532862	−1.730169	−2.571902	−0.516210	−1.806419	−2.600561
8	2.937339	0.316986	0.000000	2.956677	0.338860	0.000000	2.965742	0.315662	0.000000
1	−2.412327	1.781180	1.198435	−2.398174	1.601713	1.187634	−2.418036	1.678740	1.185840
1	0.503601	2.966011	1.265617	0.402434	2.973986	1.231825	0.418984	2.973885	1.259626
1	−0.382769	2.027970	2.583899	−0.409278	2.017109	2.561400	−0.436512	2.028621	2.574498
1	−2.412327	1.781180	−1.198435	−2.398174	1.601713	−1.187634	−2.418036	1.678740	−1.185840
1	0.503601	2.966011	−1.265617	0.402434	2.973986	−1.231825	0.418984	2.973885	−1.259626
1	−0.382769	2.027970	−2.583899	−0.409278	2.017109	−2.561400	−0.436512	2.028621	−2.574498

Table S24. Optimized *coordinates* of the **1T-1** for the $(C_4H_6)_2Fe_2(CO)$ structure.

	BP86			M06−L			B3LYP		
	x	y	z	x	y	z	x	y	z
26	0.554208	−1.069875	0.000000	0.500516	−1.119495	0.000000	0.194603	−1.663022	0.000000
26	−0.461257	0.862924	0.000000	−0.497999	0.942955	0.000000	−0.000524	0.981425	0.000000
6	0.961821	−1.416288	1.968003	0.930174	−1.460584	1.981989	0.327343	−2.046017	1.994087
6	0.961821	−1.416288	−1.968003	0.930174	−1.460584	−1.981989	0.327343	−2.046017	−1.994087
6	−0.457993	−1.427943	1.762816	−0.477058	−1.392886	1.792072	−0.801027	−1.200955	1.806275
6	−0.457993	−1.427943	−1.762816	−0.477058	−1.392886	−1.792072	−0.801027	−1.200955	−1.806275
6	−1.260937	−0.203250	1.682692	−1.223220	−0.151225	1.681576	−0.799630	0.248881	1.851213
6	−1.260937	−0.203250	−1.682692	−1.223220	−0.151225	−1.681576	−0.799630	0.248881	−1.851213
6	−0.768873	1.107106	1.999248	−0.705730	1.141026	1.998632	0.327343	1.074585	2.068416
6	−0.768873	1.107106	−1.999248	−0.705730	1.141026	−1.998632	0.327343	1.074585	−2.068416
6	0.837629	2.012709	0.000000	0.917356	1.950681	0.000000	0.392684	2.692385	0.000000
8	1.804537	2.697671	0.000000	1.947608	2.512031	0.000000	0.666235	3.828100	0.000000
1	1.461110	−0.537277	2.398745	1.468888	−0.583953	2.346267	1.255735	−1.635227	2.394020
1	1.469526	−2.375200	2.145526	1.380933	−2.418071	2.230452	0.159878	−3.101774	2.207368
1	−0.995076	−2.387551	1.721130	−1.052268	−2.318801	1.753558	−1.788007	−1.663500	1.736841
1	−2.339678	−0.341388	1.516773	−2.297377	−0.264017	1.528882	−1.787156	0.719949	1.867854
1	0.131128	1.232568	2.613983	0.206611	1.223776	2.588281	1.289943	0.623184	2.312571
1	−1.510523	1.912654	2.121549	−1.427008	1.937931	2.190935	0.179418	2.069621	2.480472
1	−0.995076	−2.387551	−1.721130	−1.052268	−2.318801	−1.753558	−1.788007	−1.663500	−1.736841
1	1.461110	−0.537277	−2.398745	1.468888	−0.583953	−2.346267	1.255735	−1.635227	−2.394020
1	1.469526	−2.375200	−2.145526	1.380933	−2.418071	−2.230452	0.159878	−3.101774	−2.207368
1	−2.339678	−0.341388	−1.516773	−2.297377	−0.264017	−1.528882	−1.787156	0.719949	−1.867854
1	0.131128	1.232568	−2.613983	0.206611	1.223776	−2.588281	1.289943	0.623184	−2.312571
1	−1.510523	1.912654	−2.121549	−1.427008	1.937931	−2.190935	0.179418	2.069621	−2.480472

Table S25. Optimized coordinates of the **1T-2** for the $(C_4H_6)_2Fe_2(CO)$ structure.

	BP86			M06-L			B3LYP		
	x	y	z	x	y	z	x	y	z
6	1.677541	0.374385	0.000000	1.616583	0.459115	0.000000	1.485334	0.367425	0.000000
26	0.372196	−1.153710	0.000000	0.697856	−1.356084	0.000000	0.449309	−1.279677	0.000000
26	−0.038679	0.913366	0.000000	−0.195759	0.654408	0.000000	−0.252520	0.979594	0.000000
6	−0.901432	−1.776470	1.513629	−0.834434	−1.287985	1.400538	−0.911254	−2.074669	1.513146
6	−0.090638	2.301463	1.489876	−0.055447	2.132409	1.374537	0.069277	2.454876	1.439704
6	−0.508515	−2.906284	0.717289	−0.746077	−2.555089	0.716698	−0.395625	−3.122779	0.714076
6	−1.306986	2.354270	0.719377	−1.320372	2.156667	0.712281	−1.125754	2.716347	0.711209
6	−0.508515	−2.906284	−0.717289	−0.746077	−2.555089	−0.716698	−0.395625	−3.122779	−0.714076
6	−1.306986	2.354270	−0.719377	−1.320372	2.156667	−0.712281	−1.125754	2.716347	−0.711209
6	−0.901432	−1.776470	−1.513629	−0.834434	−1.287985	−1.400538	−0.911254	−2.074669	−1.513146
6	−0.090638	2.301463	−1.489876	−0.055447	2.132409	−1.374537	0.069277	2.454876	−1.439704
1	−1.817077	−1.210937	1.284900	−1.629931	−0.583765	1.095046	−1.812753	−1.534753	1.224595
1	−0.643225	−1.800041	2.580236	−0.682445	−1.280883	2.478402	−0.709555	−2.096099	2.582095
1	0.092432	−3.694221	1.197269	−0.575026	−3.481520	1.263698	0.241772	−3.867545	1.195231
1	0.092432	−3.694221	−1.197269	−0.575026	−3.481520	−1.263698	0.241772	−3.867545	−1.195231
1	−1.817077	−1.210937	−1.284900	−1.629931	−0.583765	−1.095046	−1.812753	−1.534753	−1.224595
1	−0.643225	−1.800041	−2.580236	−0.682445	−1.280883	−2.478402	−0.709555	−2.096099	−2.582095
8	2.876420	0.450214	0.000000	2.700966	0.944050	0.000000	2.635877	0.661146	0.000000
1	−2.254774	2.088228	1.210794	−2.248348	1.945003	1.243714	−2.091313	2.670383	1.219556
1	0.773553	2.939023	1.253559	0.742379	2.814341	1.078631	1.019212	2.903922	1.154237
1	−0.179507	2.040532	2.552029	−0.049523	1.879060	2.434135	−0.024996	2.235656	2.502741
1	−2.254774	2.088228	−1.210794	−2.248348	1.945003	−1.243714	−2.091313	2.670383	−1.219556
1	0.773553	2.939023	−1.253559	0.742379	2.814341	−1.078631	1.019212	2.903922	−1.154237
1	−0.179507	2.040532	−2.552029	−0.049523	1.879060	−2.434135	−0.024996	2.235656	−2.502741

Table S26. Optimized coordinates of the **1Q-1** for the $(C_4H_6)_2Fe_2(CO)$ structure.

	BP86			M06-L			B3LYP		
	x	y	z	x	y	z	x	y	z
26	−0.988254	0.677132	−0.528294	−0.964984	0.722555	−0.561272	0.451174	−1.339987	0.000000
26	0.728197	−0.412610	0.438943	0.714403	−0.434050	0.443952	−0.450390	0.940111	0.000000
6	−2.397026	−0.824681	−0.889216	−2.464015	−0.691679	−0.819204	0.891383	−1.530680	2.075440
6	0.011295	2.414722	−0.907388	0.208430	2.342019	−0.921199	0.891383	−1.530680	−2.075440
6	−2.357986	−0.410463	0.478464	−2.378000	−0.208253	0.512747	−0.494599	−1.319194	1.900337
6	0.367131	2.114158	0.453841	0.546321	2.007429	0.432497	−0.494599	−1.319194	−1.900337
6	−1.280024	−0.777444	1.402839	−1.296344	−0.585742	1.414063	−1.177977	−0.044170	1.780187
6	1.551731	1.415667	0.938015	1.707137	1.290735	0.902070	−1.177977	−0.044170	−1.780187
6	−0.394884	−1.901473	1.231116	−0.525653	−1.782901	1.298738	−0.607421	1.242168	2.019737
6	2.464079	0.726309	0.089231	2.535559	0.539365	0.041724	−0.607421	1.242168	−2.019737
6	1.328914	−1.515496	−0.770595	1.181221	−1.619411	−0.761250	0.878964	2.065546	0.000000
8	1.706977	−2.232761	−1.634204	1.447643	−2.370659	−1.622219	1.814581	2.769736	0.000000
1	−1.845108	−1.712316	−1.225822	−1.964813	−1.621193	−1.097044	1.546277	−0.706572	2.356357
1	−3.277372	−0.562858	−1.490101	−3.343473	−0.446430	−1.409301	1.242737	−2.527886	2.333757
1	−3.158262	0.240390	0.866128	−3.122860	0.499006	0.880958	−1.138657	−2.204986	1.878356
1	−1.299637	−0.275030	2.380787	−1.254800	−0.037374	2.355447	−2.261449	−0.116400	1.664153
1	−0.689502	−2.708845	0.548324	−0.892008	−2.584066	0.658866	0.333766	1.321903	2.562008
1	0.163611	−2.244081	2.117434	0.007265	−2.128329	2.187171	−1.296402	2.068006	2.208535
1	−0.248815	2.576553	1.242968	−0.038425	2.492034	1.218710	−1.138657	−2.204986	−1.878356
1	0.756533	2.272312	−1.703080	0.955032	2.171412	−1.697506	1.546277	−0.706572	−2.356357
1	−0.702637	3.234398	−1.077210	−0.412778	3.226117	−1.071494	1.242737	−2.527886	−2.333757
1	1.729852	1.434513	2.024067	1.901597	1.301911	1.974813	−2.261449	−0.116400	−1.664153
1	2.542509	0.977884	−0.975568	2.583636	0.772137	−1.020414	0.333766	1.321903	−2.562008
1	3.375126	0.303812	0.530993	3.427649	0.069545	0.446756	−1.296402	2.068006	−2.208535

Table S27. Optimized coordinates of the **1Q-2** for *the* $(C_4H_6)_2Fe_2(CO)$ structure.

	BP86			M06-L			B3LYP		
	x	y	z	x	y	z	x	y	z
6	−0.222024	1.684836	0.002670	−0.225342	1.700372	−0.025294	−0.265970	1.713621	0.017347
26	1.303635	0.442165	0.102459	1.329594	0.462065	0.117527	1.340393	0.464660	0.121223
26	−0.903788	−0.030344	−0.035745	−0.896428	−0.028864	−0.007909	−0.934838	−0.007732	−0.087516
6	3.045926	−0.341054	1.000802	3.162591	−0.267670	0.855143	3.008097	−0.475505	1.092418
6	−1.879517	−0.625255	1.700055	−2.047485	−0.441259	1.668151	−1.848690	−0.772989	1.721224
6	3.076968	−0.499220	−0.417327	3.010070	−0.534532	−0.526895	3.144540	−0.477694	−0.316784
6	−2.308488	−1.429284	0.595296	−2.340772	−1.358334	0.626653	−2.282332	−1.496135	0.586622
6	1.960780	−1.052168	−1.153512	1.844951	−1.187710	−1.050822	2.111612	−0.929380	−1.209791
6	−2.723113	−0.858798	−0.663731	−2.627432	−0.935485	−0.710303	−2.798760	−0.882112	−0.604976
6	0.779986	−1.603986	−0.515940	0.785519	−1.657251	−0.211105	0.869842	−1.515908	−0.778161
6	−2.746258	0.545718	−0.904995	−2.639909	0.424541	−1.092511	−2.936464	0.511137	−0.781515
1	2.563648	−1.091683	1.645935	2.784032	−0.971091	1.599064	2.444911	−1.254188	1.609727
1	3.843056	0.238178	1.481876	4.009740	0.329062	1.180165	3.774625	0.012615	1.690164
1	3.846920	0.031574	−0.996652	3.682629	−0.057005	−1.238995	3.971851	0.077959	−0.761847
1	1.954987	−0.915137	−2.244510	1.697897	−1.180038	−2.129329	2.229215	−0.686158	−2.266984
1	0.905367	−2.215274	0.391448	1.032426	−2.106552	0.753763	0.883050	−2.205457	0.069233
1	0.038788	−2.021487	−1.217005	−0.022365	−2.178532	−0.729728	0.209172	−1.842765	−1.584707
8	−0.357983	2.880367	−0.022217	−0.414829	2.876098	−0.069664	−0.389313	2.891200	0.019513
1	−2.085409	−2.505918	0.602170	−2.099513	−2.411371	0.768923	−2.022007	−2.552915	0.513302
1	−2.387258	0.315747	1.958219	−2.579209	0.507938	1.748685	−2.330586	0.156631	2.024580
1	−1.399928	−1.125313	2.550006	−1.678672	−0.829778	2.612855	−1.341296	−1.305707	2.521630
1	−2.771453	−1.543927	−1.523189	−2.578077	−1.699390	−1.486661	−2.876000	−1.523792	−1.484415
1	−3.069348	1.257039	−0.131633	−3.027150	1.193427	−0.422757	−3.196711	1.170320	0.046246
1	−2.877096	0.901165	−1.933385	−2.698581	0.675296	−2.146856	−3.187373	0.893513	−1.767736

Table S28. Harmonic vibrational frequencies (in cm^{-1}) and infrared intensities (in parentheses, in km/mol) for the structure **2S-1** of $(C_4H_6)_2Fe_2(CO)_2$ structure.

BP86	M06-L	B3LYP	BP86	M06-L	B3LYP	BP86	M06-L	B3LYP
63(0)	54(0)	58(0)	564(55)	581(21)	571(37)	1163(2)	1193(3)	1199(2)
71(0)	68(0)	64(1)	579(14)	592(55)	583(77)	1193(7)	1231(13)	1236(17)
91(1)	87(1)	88(1)	644(3)	672(7)	661(6)	1327(3)	1365(1)	1382(2)
117(1)	114(1)	118(1)	655(9)	683(5)	684(3)	1348(0)	1385(1)	1403(0)
132(1)	135(1)	122(1)	709(19)	731(5)	737(3)	1410(4)	1444(4)	1461(5)
150(1)	158(3)	148(0)	741(8)	752(7)	759(4)	1414(7)	1452(5)	1474(6)
158(2)	174(1)	156(2)	758(6)	773(9)	773(23)	1425(2)	1466(1)	1479(5)
198(1)	200(1)	191(1)	791(24)	817(20)	829(24)	1438(5)	1493(4)	1493(4)
216(2)	218(1)	213(2)	851(4)	868(5)	881(6)	1459(3)	1501(6)	1518(8)
262(1)	266(2)	257(1)	855(8)	879(3)	900(17)	1468(8)	1512(11)	1524(15)
324(4)	335(2)	326(1)	860(4)	886(2)	905(4)	1793(491)	1883(602)	1875(656)
345(1)	363(0)	346(1)	893(3)	898(9)	912(11)	1942(1037)	2035(1173)	2052(1213)
349(2)	376(6)	364(4)	902(17)	925(12)	939(6)	2392(2)	2624(0)	2597(2)
394(3)	418(2)	386(5)	913(6)	935(10)	958(6)	2913(7)	3008(2)	3055(1)
398(4)	424(2)	393(4)	925(1)	950(2)	960(4)	3036(17)	3089(36)	3117(10)
408(4)	431(15)	412(6)	934(5)	967(5)	975(3)	3039(12)	3100(28)	3125(9)
431(11)	455(20)	434(29)	939(3)	973(3)	985(2)	3093(2)	3157(6)	3166(2)
450(0)	467(6)	460(15)	1006(5)	1039(5)	1045(6)	3099(0)	3158(2)	3176(4)
456(2)	473(3)	471(1)	1028(5)	1059(6)	1066(1)	3102(4)	3163(12)	3178(0)
475(9)	486(6)	478(2)	1031(4)	1067(4)	1068(10)	3108(10)	3169(23)	3184(8)
484(5)	503(3)	484(8)	1043(4)	1078(7)	1078(6)	3117(5)	3180(42)	3190(5)
495(4)	513(17)	493(3)	1099(4)	1145(5)	1149(3)	3120(9)	3181(6)	3201(9)
531(20)	542(34)	523(19)	1150(5)	1173(1)	1164(3)	3126(8)	3192(17)	3203(8)
560(19)	562(63)	558(84)	1153(4)	1188(8)	1194(10)	3129(7)	3192(27)	3210(7)

Table S29. Harmonic vibrational frequencies (in cm^{-1}) and infrared intensities (in parentheses, in km/mol) for the structure **2S-2** of $(C_4H_6)_2Fe_2(CO)_2$ structure.

BP86	M06-L	B3LYP	BP86	M06-L	B3LYP	BP86	M06−L	B3LYP
38(0)	49(2)	43(0)	532(0)	543(0)	540(0)	1234(14)	1268(25)	1275(55)
44(2)	57(0)	51(3)	539(6)	545(13)	540(82)	1235(0)	1269(0)	1277(0)
50(0)	64(0)	59(0)	554(9)	571(16)	565(0)	1357(2)	1392(0)	1414(2)
79(2)	87(2)	92(1)	612(37)	610(58)	580(11)	1357(0)	1393(0)	1414(0)
93(2)	92(2)	103(2)	650(0)	678(0)	696(0)	1413(16)	1450(14)	1461(42)
101(0)	107(0)	105(0)	659(0)	684(0)	697(0)	1413(0)	1450(0)	1462(0)
114(0)	108(0)	114(0)	667(15)	700(13)	712(16)	1463(0)	1512(0)	1524(86)
186(6)	178(6)	174(10)	715(36)	729(31)	723(3)	1464(9)	1512(11)	1525(0)
200(0)	207(0)	183(0)	847(7)	866(3)	875(8)	1468(23)	1523(41)	1532(0)
241(0)	251(0)	234(0)	850(0)	868(0)	888(0)	1469(0)	1524(0)	1532(10)
245(3)	258(3)	235(10)	855(17)	873(31)	892(0)	1761(883)	1840(1043)	1811(1172)
286(0)	293(0)	294(0)	855(0)	876(0)	897(24)	1783(0)	1865(0)	1857(0)
361(35)	375(39)	359(0)	880(29)	903(43)	923(20)	3021(0)	3079(0)	3099(0)
380(0)	394(0)	372(47)	884(0)	907(0)	926(0)	3021(2)	3080(0)	3099(4)
385(4)	395(4)	382(5)	892(8)	924(5)	939(30)	3030(10)	3087(22)	3109(17)
397(0)	407(0)	401(0)	894(0)	925(0)	940(0)	3030(0)	3087(0)	3109(0)
397(1)	420(0)	402(4)	927(0)	954(0)	988(0)	3092(3)	3156(0)	3169(3)
404(0)	423(0)	405(2)	931(2)	958(2)	990(1)	3092(0)	3156(9)	3169(0)
412(2)	446(5)	417(0)	1023(17)	1060(25)	1062(19)	3105(0)	3170(55)	3183(18)
423(0)	452(0)	435(0)	1025(0)	1062(0)	1063(0)	3105(17)	3170(0)	3183(0)
447(0)	467(0)	439(0)	1041(0)	1076(9)	1082(0)	3150(8)	3223(0)	3227(10)
457(0)	474(0)	460(0)	1041(10)	1076(0)	1083(10)	3150(0)	3223(35)	3227(0)
484(2)	494(2)	501(5)	1165(0)	1205(0)	1212(0)	3152(2)	3224(8)	3229(3)
530(0)	542(0)	527(51)	1166(8)	1205(8)	1212(10)	3152(0)	3225(0)	3230(0)

Table S30. Harmonic vibrational frequencies (in cm^{-1}) and infrared intensities (in parentheses, in km/mol) for the structure **2S-3** of $(C_4H_6)_2Fe_2(CO)_2$ structure.

BP86	M06-L	B3LYP	BP86	M06-L	B3LYP	BP86	M06-L	B3LYP
45(6)	44(0)	−117(7)	549(0)	563(3)	560(12)	1225(7)	1256(15)	1269(16)
48(0)	51(4)	48(0)	562(1)	575(23)	571(26)	1232(1)	1265(2)	1274(3)
60(0)	80(0)	53(0)	591(26)	584(8)	578(1)	1348(1)	1382(2)	1406(1)
83(0)	83(8)	73(1)	602(4)	605(2)	584(26)	1349(1)	1384(1)	1407(3)
92(9)	94(0)	94(1)	706(3)	713(5)	703(18)	1409(2)	1444(3)	1442(11)
103(1)	101(1)	110(0)	734(0)	741(0)	721(0)	1414(6)	1449(5)	1444(10)
108(0)	112(0)	133(1)	753(2)	760(2)	752(13)	1461(8)	1501(13)	1511(32)
156(0)	158(0)	141(4)	754(13)	761(13)	759(0)	1464(1)	1507(0)	1513(2)
157(1)	159(1)	146(0)	831(8)	843(4)	862(7)	1484(22)	1529(39)	1530(17)
246(1)	264(1)	245(0)	844(2)	856(1)	863(0)	1492(7)	1540(4)	1530(1)
279(2)	290(2)	253(0)	864(7)	874(8)	879(9)	1925(424)	1998(472)	2005(1280)
295(1)	310(1)	304(0)	865(7)	881(2)	887(27)	1955(1040)	2039(1188)	2037(1211)
335(8)	340(9)	315(3)	869(2)	884(5)	920(0)	3021(11)	3088(47)	3131(0)
344(3)	347(2)	326(1)	889(3)	900(1)	920(2)	3022(0)	3089(0)	3131(4)
376(4)	393(5)	394(8)	903(0)	909(1)	950(9)	3044(3)	3115(6)	3139(4)
393(0)	407(0)	416(1)	909(10)	914(12)	953(1)	3045(4)	3115(9)	3139(3)
437(1)	451(1)	431(32)	919(2)	934(3)	971(0)	3064(6)	3128(25)	3159(0)
451(17)	466(13)	442(0)	931(0)	945(1)	987(4)	3064(6)	3128(14)	3163(2)
465(6)	472(13)	462(5)	1013(2)	1038(4)	1047(0)	3070(2)	3138(37)	3176(0)
474(1)	492(1)	483(10)	1015(2)	1041(3)	1048(1)	3071(12)	3139(4)	3178(16)
479(0)	505(1)	499(0)	1042(0)	1073(0)	1085(0)	3121(8)	3188(14)	3219(1)
516(9)	533(11)	515(1)	1043(7)	1074(5)	1086(6)	3122(5)	3188(14)	3220(4)
522(49)	540(50)	536(50)	1170(3)	1205(5)	1213(6)	3151(5)	3221(17)	3232(3)
545(0)	547(5)	559(54)	1174(0)	1210(0)	1218(1)	3152(2)	3221(3)	3233(1)

Table S31. Harmonic vibrational frequencies (in cm^{-1}) and infrared intensities (in parentheses, in km/mol) for the structure **2T-1** of $(C_4H_6)_2Fe_2(CO)_2$ structure.

BP86	M06-L	B3LYP	BP86	M06-L	B3LYP	BP86	M06−L	B3LYP
40(1)	37(1)	30(1)	518(3)	515(4)	516(32)	1218(13)	1252(17)	1254(24)
53(1)	56(1)	51(1)	531(14)	530(20)	560(192)	1220(0)	1254(0)	1255(0)
74(0)	67(0)	70(0)	584(5)	598(8)	608(6)	1355(0)	1389(0)	1407(0)
80(0)	86(0)	81(0)	594(1)	610(0)	618(6)	1355(1)	1389(0)	1407(0)
100(0)	102(1)	88(2)	654(5)	666(4)	702(1)	1420(10)	1457(12)	1470(0)
112(0)	123(0)	124(0)	672(1)	690(0)	707(79)	1421(0)	1457(0)	1473(41)
126(0)	126(0)	124(0)	687(4)	704(1)	716(1)	1462(6)	1506(1)	1517(12)
164(1)	155(1)	148(4)	692(1)	704(1)	722(139)	1463(10)	1506(12)	1517(6)
186(1)	187(0)	185(0)	841(13)	862(9)	875(6)	1468(10)	1515(11)	1524(5)
199(0)	199(0)	194(0)	847(2)	868(4)	881(2)	1468(1)	1517(0)	1524(11)
240(3)	245(3)	220(5)	855(9)	870(2)	888(11)	1842(1078)	1931(1315)	1936(1475)
267(0)	270(0)	271(0)	856(4)	870(9)	892(15)	1858(65)	1951(62)	1961(68)
330(9)	325(8)	274(0)	876(1)	894(7)	917(0)	3057(0)	3115(5)	3135(0)
351(0)	355(0)	355(1)	877(0)	896(0)	917(18)	3057(2)	3115(17)	3136(3)
375(5)	377(6)	363(8)	904(9)	930(15)	938(0)	3061(5)	3119(17)	3141(0)
376(8)	397(4)	369(0)	905(0)	931(0)	947(16)	3061(0)	3119(3)	3141(7)
385(2)	404(2)	397(33)	913(1)	940(1)	966(1)	3097(2)	3161(6)	3175(1)
396(0)	413(0)	398(1)	914(2)	941(1)	967(1)	3097(2)	3161(2)	3176(0)
413(13)	437(15)	445(0)	1029(15)	1063(22)	1065(0)	3114(0)	3176(0)	3190(0)
440(1)	465(2)	460(1)	1030(0)	1064(2)	1074(13)	3114(8)	3176(31)	3190(9)
447(3)	470(0)	462(1)	1036(11)	1070(8)	1074(0)	3153(0)	3223(3)	3230(7)
450(1)	476(0)	468(17)	1037(0)	1070(1)	1077(21)	3154(7)	3223(18)	3230(1)
461(1)	477(16)	473(4)	1164(0)	1201(0)	1203(0)	3155(2)	3225(4)	3233(1)
473(13)	480(2)	507(2)	1164(8)	1201(9)	1203(10)	3155(1)	3225(13)	3233(9)

Table S32. Harmonic vibrational frequencies (in cm^{-1}) and infrared intensities (in parentheses, in km/mol) for the structure **2T-2** of $(C_4H_6)_2Fe_2(CO)_2$ structure.

BP86	M06-L	B3LYP	BP86	M06-L	B3LYP	BP86	M06−L	B3LYP
30(0)	39(0)	29(0)	565(33)	560(52)	562(58)	1153(4)	1194(5)	1198(6)
51(1)	59(1)	46(1)	590(47)	598(62)	614(58)	1194(10)	1235(11)	1241(16)
73(1)	77(0)	84(0)	620(5)	641(6)	628(15)	1322(2)	1364(0)	1374(2)
98(2)	107(1)	95(2)	665(3)	683(3)	663(3)	1348(0)	1384(0)	1405(0)
106(2)	112(2)	108(1)	709(1)	736(8)	728(8)	1395(4)	1442(1)	1440(2)
133(2)	130(1)	123(2)	724(9)	739(1)	731(2)	1406(4)	1462(3)	1471(5)
150(2)	153(1)	142(1)	736(8)	755(6)	745(11)	1427(3)	1463(5)	1478(13)
183(0)	194(1)	179(0)	770(5)	781(4)	778(6)	1430(9)	1476(7)	1495(4)
219(1)	219(2)	202(1)	833(6)	853(6)	860(22)	1458(4)	1504(5)	1522(14)
233(1)	237(1)	224(1)	848(8)	871(4)	889(22)	1468(7)	1510(10)	1524(8)
301(1)	310(1)	290(2)	876(2)	892(6)	895(5)	1908(485)	1981(592)	1998(803)
315(2)	324(0)	307(0)	877(7)	898(6)	916(6)	1957(1296)	2043(1346)	2053(1246)
373(1)	386(1)	359(5)	906(10)	923(10)	925(9)	2515(6)	2744(8)	2737(13)
387(4)	398(2)	365(3)	920(1)	942(1)	952(2)	2561(3)	2802(2)	2773(5)
389(4)	412(2)	373(0)	926(3)	951(5)	967(10)	3046(8)	3106(30)	3129(5)
405(4)	427(3)	406(1)	933(4)	967(6)	974(2)	3048(12)	3110(20)	3134(6)
421(1)	444(12)	417(7)	986(8)	1019(15)	1007(21)	3098(2)	3162(6)	3172(4)
434(11)	447(1)	448(5)	1017(11)	1042(8)	1020(6)	3100(4)	3170(10)	3178(2)
449(4)	464(4)	453(1)	1025(2)	1058(3)	1048(6)	3113(1)	3177(1)	3180(1)
458(4)	487(5)	458(4)	1040(1)	1060(1)	1069(3)	3118(5)	3179(14)	3188(6)
480(16)	491(7)	479(6)	1044(8)	1069(4)	1074(2)	3121(4)	3186(24)	3198(2)
484(3)	499(1)	504(3)	1058(4)	1074(12)	1076(9)	3130(8)	3198(25)	3202(9)
499(5)	513(11)	508(12)	1097(5)	1148(5)	1143(4)	3134(6)	3198(17)	3214(7)
529(13)	536(10)	521(14)	1134(3)	1181(4)	1177(4)	3140(3)	3207(9)	3226(3)

Table S33. Harmonic vibrational frequencies (in cm^{-1}) and infrared intensities (in parentheses, in km/mol) for the structure **2T-3** of $(C_4H_6)_2Fe_2(CO)_2$ structure.

BP86	M06-L	B3LYP	BP86	M06-L	B3LYP	BP86	M06-L	B3LYP
35(0)	43(0)	39(0)	544(17)	552(3)	551(8)	1225(5)	1256(9)	1267(21)
52(0)	53(0)	49(0)	553(0)	568(20)	563(22)	1232(1)	1263(2)	1276(1)
62(0)	54(0)	51(0)	579(24)	577(0)	579(0)	1348(0)	1383(1)	1404(0)
80(0)	99(1)	85(9)	593(5)	601(5)	594(8)	1349(1)	1384(1)	1405(1)
101(2)	110(0)	96(0)	699(4)	702(5)	715(4)	1404(0)	1440(1)	1451(12)
122(0)	121(0)	105(0)	716(0)	712(1)	725(1)	1408(7)	1442(6)	1459(9)
122(6)	123(4)	110(0)	733(0)	746(5)	757(9)	1458(9)	1494(8)	1504(17)
143(1)	145(1)	141(1)	738(4)	747(0)	757(0)	1462(1)	1500(1)	1512(1)
158(4)	158(2)	148(1)	833(9)	856(4)	875(7)	1477(17)	1523(31)	1540(34)
250(1)	258(0)	216(0)	838(2)	858(1)	882(7)	1482(6)	1529(2)	1545(3)
269(1)	272(2)	268(5)	864(10)	881(6)	900(5)	1925(656)	2000(725)	1995(924)
282(1)	279(1)	271(0)	872(4)	885(9)	905(4)	1950(1043)	2032(1214)	2027(1268)
341(7)	343(2)	314(4)	882(4)	896(4)	925(0)	3045(8)	3098(38)	3102(10)
344(3)	344(7)	326(2)	899(0)	901(0)	926(10)	3045(0)	3098(0)	3102(0)
394(1)	396(1)	366(1)	900(2)	912(5)	938(1)	3061(0)	3128(3)	3140(1)
395(0)	397(3)	371(4)	902(8)	915(0)	946(1)	3062(3)	3128(10)	3140(4)
431(5)	443(16)	417(16)	914(3)	928(1)	948(1)	3073(3)	3132(15)	3151(2)
439(0)	451(0)	434(0)	923(0)	941(0)	960(0)	3073(5)	3132(13)	3151(3)
449(13)	466(9)	440(19)	1011(2)	1032(3)	1048(4)	3078(1)	3143(38)	3160(1)
475(0)	494(0)	478(0)	1012(2)	1034(2)	1049(1)	3079(12)	3144(5)	3161(15)
483(0)	514(1)	505(1)	1043(0)	1078(1)	1088(0)	3133(3)	3196(11)	3204(4)
509(6)	526(5)	518(9)	1046(6)	1078(2)	1089(3)	3133(3)	3196(8)	3204(5)
523(50)	534(43)	537(39)	1172(1)	1207(2)	1211(2)	3163(1)	3234(1)	3242(5)
544(0)	543(32)	543(50)	1174(0)	1212(1)	1217(1)	3163(3)	3234(18)	3243(1)

Table S34. Harmonic vibrational frequencies (in cm^{-1}) and infrared intensities (in parentheses, in km/mol) for the structure **2Q-1** of $(C_4H_6)_2Fe_2(CO)_2$ structure.

BP86	M06-L	B3LYP	BP86	M06-L	B3LYP	BP86	M06-L	B3LYP
50(0)	48(0)	51(0)	496(12)	508(3)	501(15)	1237(15)	1271(21)	1282(38)
75(0)	62(1)	64(1)	526(29)	521(14)	536(11)	1243(9)	1276(12)	1288(17)
76(1)	73(0)	79(0)	551(7)	563(7)	570(8)	1354(0)	1389(0)	1408(0)
87(0)	77(0)	89(0)	557(2)	568(2)	572(2)	1356(2)	1391(4)	1410(2)
122(0)	116(0)	109(0)	669(1)	680(1)	690(1)	1397(11)	1435(10)	1442(35)
127(1)	123(1)	122(1)	684(2)	695(3)	700(4)	1401(17)	1438(13)	1448(23)
150(0)	154(0)	153(0)	704(0)	709(0)	735(1)	1465(21)	1510(24)	1524(55)
157(0)	156(0)	158(0)	727(1)	732(2)	750(3)	1469(4)	1516(1)	1529(12)
211(0)	186(0)	176(3)	813(2)	821(0)	842(2)	1483(28)	1522(32)	1537(35)
247(0)	254(0)	228(2)	820(53)	829(62)	846(66)	1484(41)	1523(59)	1538(44)
250(0)	256(0)	257(0)	836(6)	853(7)	865(4)	1815(454)	1913(549)	1922(628)
268(0)	268(1)	261(2)	841(4)	856(3)	869(10)	1962(1045)	2047(1186)	2045(1188)
308(17)	296(13)	294(3)	865(1)	875(0)	904(8)	3055(1)	3117(24)	3134(1)
324(1)	322(2)	303(3)	875(17)	881(14)	918(4)	3057(2)	3118(15)	3136(1)
331(0)	333(5)	316(3)	891(0)	897(1)	925(0)	3059(5)	3122(1)	3137(5)
332(2)	341(1)	328(0)	893(1)	897(5)	932(1)	3059(4)	3122(2)	3138(6)
371(10)	388(7)	360(11)	903(0)	922(3)	945(1)	3064(0)	3124(4)	3141(0)
412(3)	415(6)	390(5)	906(2)	922(0)	950(3)	3064(4)	3124(18)	3141(5)
417(3)	420(0)	421(3)	1009(2)	1034(2)	1048(1)	3073(0)	3143(39)	3153(0)
423(1)	428(1)	425(1)	1009(1)	1034(3)	1048(3)	3075(16)	3143(1)	3155(16)
438(0)	452(6)	428(5)	1046(1)	1077(1)	1086(1)	3145(0)	3213(0)	3225(0)
466(16)	469(41)	469(25)	1052(8)	1083(7)	1092(12)	3146(6)	3213(19)	3225(7)
488(20)	484(25)	473(64)	1175(1)	1209(2)	1216(0)	3155(0)	3225(1)	3235(0)
496(5)	494(6)	489(3)	1179(6)	1214(7)	1222(6)	3156(7)	3225(23)	3235(6)

Table S35. Harmonic vibrational frequencies (in cm^{-1}) and infrared intensities (in parentheses, in km/mol) for the structure **2Q-2** of $(C_4H_6)_2Fe_2(CO)_2$ structure.

BP86	M06-L	B3LYP	BP86	M06-L	B3LYP	BP86	M06−L	B3LYP
20(0)	27(1)	21(0)	557(11)	533(35)	547(40)	1203(4)	1232(5)	1237(7)
53(1)	48(1)	42(0)	561(26)	572(18)	576(3)	1253(24)	1284(25)	1293(42)
68(0)	72(0)	73(1)	582(16)	578(25)	586(46)	1342(3)	1379(6)	1394(7)
80(0)	80(1)	76(1)	599(17)	635(4)	629(7)	1364(1)	1397(0)	1419(1)
83(1)	90(1)	79(0)	631(39)	648(37)	645(54)	1414(4)	1453(8)	1463(28)
88(0)	98(0)	103(1)	655(6)	690(1)	694(0)	1417(17)	1459(7)	1474(6)
102(1)	108(1)	111(0)	661(4)	702(9)	706(2)	1434(4)	1474(6)	1481(5)
111(1)	134(0)	117(0)	713(0)	726(3)	729(6)	1479(12)	1522(13)	1525(15)
119(0)	154(1)	147(1)	801(11)	812(19)	840(16)	1487(51)	1538(57)	1542(94)
193(0)	206(1)	177(0)	819(10)	815(10)	851(13)	1497(17)	1545(28)	1560(11)
243(3)	261(1)	212(2)	841(7)	858(5)	870(12)	1912(798)	1966(820)	1974(952)
258(1)	265(3)	230(3)	863(9)	871(10)	885(12)	1948(706)	2020(744)	2035(855)
280(0)	303(1)	283(0)	891(7)	912(7)	916(2)	2979(10)	3024(27)	3052(10)
317(4)	314(3)	305(2)	898(2)	926(1)	944(6)	3039(4)	3096(9)	3116(4)
350(1)	366(4)	343(5)	913(9)	935(11)	951(16)	3050(4)	3110(6)	3120(8)
371(3)	382(2)	354(1)	930(2)	964(12)	966(2)	3053(3)	3124(11)	3123(3)
385(4)	397(10)	382(5)	945(4)	965(1)	986(13)	3071(3)	3131(8)	3151(3)
397(3)	405(4)	391(1)	948(11)	976(6)	987(7)	3092(1)	3155(5)	3168(2)
411(8)	411(7)	408(9)	992(5)	1023(6)	1027(6)	3095(1)	3161(7)	3169(2)
423(2)	434(2)	413(2)	1032(3)	1062(4)	1065(2)	3107(3)	3175(10)	3184(3)
457(6)	464(3)	460(4)	1050(6)	1082(7)	1088(9)	3114(12)	3183(35)	3191(14)
487(1)	483(2)	490(8)	1075(3)	1103(3)	1111(3)	3157(3)	3226(10)	3236(2)
504(3)	496(4)	494(8)	1145(7)	1182(7)	1188(8)	3160(6)	3226(17)	3237(6)
514(7)	507(4)	509(2)	1188(7)	1225(8)	1233(8)	3164(2)	3234(7)	3238(1)

Table S36. Harmonic vibrational frequencies (in cm^{-1}) and infrared intensities (in parentheses, in km/mol) for the structure **2Q−3** of $(C_4H_6)_2Fe_2(CO)_2$ structure.

BP86	M06-L	B3LYP	BP86	M06-L	B3LYP	BP86	M06-L	B3LYP
−39(0)	24(1)	−2(1)	523(0)	508(0)	523(7)	1237(20)	1278(36)	1277(47)
1(2)	37(0)	21(0)	525(8)	508(8)	527(0)	1238(0)	1279(0)	1279(0)
46(0)	65(1)	42(15)	561(0)	570(0)	579(0)	1360(0)	1395(0)	1412(0)
53(10)	74(4)	50(1)	568(1)	574(4)	588(2)	1360(1)	1396(0)	1412(1)
83(1)	85(1)	87(1)	645(33)	653(47)	661(85)	1417(0)	1455(0)	1465(25)
95(0)	94(0)	95(0)	653(0)	660(0)	671(0)	1417(15)	1456(14)	1466(0)
95(0)	100(0)	96(0)	663(0)	686(0)	693(0)	1476(34)	1530(0)	1531(66)
131(3)	135(5)	118(7)	667(1)	689(0)	699(2)	1477(0)	1530(20)	1532(0)
152(0)	160(0)	138(0)	822(22)	820(9)	846(11)	1478(0)	1533(68)	1538(19)
186(0)	198(0)	150(0)	825(0)	823(0)	850(0)	1478(14)	1534(0)	1538(0)
208(21)	213(61)	211(30)	830(0)	825(0)	858(0)	1842(1002)	1911(1243)	1949(0)
249(39)	250(39)	229(0)	832(5)	827(16)	859(9)	1858(0)	1937(0)	1951(1295)
253(0)	268(0)	239(0)	873(4)	905(10)	913(2)	3067(0)	3125(18)	3149(0)
258(0)	273(0)	247(2)	874(0)	905(0)	915(0)	3067(0)	3125(0)	3149(0)
275(3)	280(4)	258(35)	894(3)	923(4)	922(2)	3067(5)	3127(0)	3150(4)
300(0)	312(0)	309(0)	895(0)	924(0)	923(0)	3068(0)	3127(2)	3151(0)
362(8)	377(2)	338(2)	924(0)	961(0)	970(0)	3091(0)	3161(0)	3167(0)
375(0)	396(0)	374(0)	925(2)	964(2)	972(2)	3091(2)	3161(6)	3167(2)
382(9)	398(18)	382(7)	1026(10)	1060(12)	1060(8)	3104(0)	3174(0)	3182(0)
422(0)	432(0)	434(0)	1027(0)	1061(0)	1060(0)	3104(9)	3174(27)	3182(8)
425(0)	438(13)	438(11)	1044(0)	1080(13)	1083(0)	3161(0)	3238(9)	3242(0)
430(9)	442(0)	449(0)	1044(14)	1080(0)	1083(20)	3161(8)	3239(0)	3242(6)
437(0)	445(0)	450(0)	1178(0)	1218(0)	1220(0)	3162(4)	3239(30)	3243(6)
460(19)	464(19)	479(27)	1179(8)	1219(11)	1220(10)	3162(0)	3239(0)	3243(0)

Table S37. Harmonic vibrational frequencies (in cm^{-1}) and infrared intensities (in parentheses, in km/mol) for the structure **1S−1** of $(C_4H_6)_2Fe_2(CO)$ structure.

BP86	M06-L	B3LYP	BP86	M06-L	B3LYP	BP86	M06-L	B3LYP
75(0)	66(0)	13(0)	601(5)	602(11)	607(3)	1200(1)	1234(3)	1268(11)
77(0)	70(0)	75(0)	627(0)	641(1)	663(1)	1327(1)	1364(0)	1395(2)
89(0)	76(0)	88(0)	681(1)	705(1)	732(2)	1328(0)	1365(0)	1398(0)
114(9)	103(9)	128(0)	697(0)	708(0)	733(1)	1365(3)	1411(5)	1427(26)
153(1)	158(1)	148(1)	723(5)	735(5)	766(10)	1370(3)	1416(3)	1443(13)
171(0)	159(1)	173(0)	811(6)	827(1)	835(16)	1427(10)	1470(18)	1509(49)
173(2)	171(2)	183(0)	817(16)	833(9)	836(3)	1428(12)	1471(13)	1517(4)
277(4)	286(5)	243(7)	829(2)	834(2)	861(1)	1451(11)	1492(11)	1520(1)
297(0)	316(1)	285(3)	847(29)	856(46)	877(33)	1453(4)	1495(2)	1527(36)
310(3)	328(2)	290(2)	852(3)	873(5)	887(2)	1933(901)	2011(1040)	1996(1046)
359(6)	372(6)	297(1)	861(16)	879(3)	895(45)	3022(2)	3088(14)	3095(0)
418(16)	435(1)	350(9)	862(1)	881(19)	925(7)	3023(4)	3089(12)	3095(3)
422(1)	439(41)	382(6)	877(4)	897(3)	946(4)	3042(4)	3108(20)	3122(9)
428(8)	450(0)	438(4)	896(2)	913(14)	961(2)	3043(5)	3109(18)	3123(8)
464(21)	472(25)	441(41)	899(1)	918(1)	975(0)	3078(1)	3140(27)	3151(0)
471(21)	483(24)	459(0)	994(1)	1021(1)	1043(1)	3078(11)	3141(1)	3154(3)
483(6)	500(3)	485(11)	997(2)	1023(2)	1046(2)	3097(2)	3162(47)	3167(0)
484(0)	506(16)	502(16)	1014(1)	1046(1)	1067(0)	3099(17)	3162(4)	3171(18)
494(0)	506(1)	519(14)	1020(12)	1052(13)	1073(15)	3111(0)	3185(0)	3192(0)
512(1)	515(9)	523(17)	1140(1)	1179(1)	1199(7)	3112(14)	3186(30)	3195(13)
532(28)	540(13)	549(24)	1145(4)	1185(7)	1206(9)	3131(4)	3199(5)	3207(2)
572(4)	589(4)	578(13)	1193(12)	1228(19)	1255(35)	3132(7)	3199(22)	3207(10)

Table S38. Harmonic vibrational frequencies (in cm^{-1}) and infrared intensities (in parentheses, in km/mol) for the structure **1S−2** of $(C_4H_6)_2Fe_2(CO)$ structure.

BP86	M06-L	B3LYP	BP86	M06-L	B3LYP	BP86	M06-L	B3LYP
54(0)	54(0)	59(0)	586(0)	606(0)	608(0)	1238(9)	1273(13)	1291(20)
70(1)	68(1)	69(1)	664(4)	686(2)	692(7)	1354(0)	1389(0)	1411(0)
81(0)	86(0)	83(0)	674(3)	691(3)	699(4)	1361(0)	1398(1)	1419(0)
87(0)	91(0)	88(0)	688(4)	717(2)	711(2)	1412(2)	1447(3)	1462(18)
112(1)	112(2)	119(2)	695(3)	718(2)	719(2)	1413(7)	1452(6)	1464(8)
143(1)	149(1)	150(1)	839(11)	863(1)	868(5)	1454(7)	1500(9)	1515(22)
197(1)	192(2)	178(4)	846(15)	871(3)	887(15)	1457(8)	1508(4)	1519(8)
228(0)	234(0)	231(1)	855(0)	875(15)	888(0)	1459(6)	1509(11)	1521(17)
245(0)	245(0)	248(1)	864(15)	884(22)	895(19)	1461(2)	1512(7)	1530(3)
295(3)	304(2)	300(6)	869(3)	892(13)	917(2)	1826(716)	1907(876)	1914(958)
362(9)	356(12)	318(7)	874(4)	895(0)	921(2)	3026(0)	3087(3)	3106(0)
387(9)	398(9)	363(9)	892(2)	919(1)	928(19)	3029(3)	3088(8)	3113(4)
392(4)	414(3)	384(12)	896(9)	923(10)	933(5)	3033(13)	3090(22)	3131(2)
415(3)	438(4)	417(1)	898(1)	929(0)	960(1)	3033(2)	3092(17)	3132(8)
423(1)	439(5)	418(4)	903(6)	931(6)	971(1)	3069(1)	3136(3)	3143(1)
427(0)	445(0)	424(1)	1015(19)	1050(28)	1054(17)	3078(2)	3138(5)	3154(2)
445(6)	465(0)	438(0)	1017(5)	1052(5)	1056(4)	3084(9)	3152(27)	3161(8)
452(1)	471(3)	451(0)	1038(3)	1073(2)	1079(3)	3092(11)	3153(28)	3170(10)
457(1)	488(1)	464(0)	1045(4)	1079(4)	1088(5)	3132(9)	3199(27)	3221(8)
474(8)	493(9)	476(16)	1158(5)	1199(5)	1203(6)	3133(5)	3200(11)	3222(7)
547(10)	543(13)	547(17)	1165(9)	1206(10)	1217(9)	3145(6)	3214(21)	3222(6)
555(5)	562(7)	582(6)	1225(6)	1263(11)	1270(19)	3147(1)	3216(3)	3224(1)

Table S39. Harmonic vibrational frequencies (in cm^{-1}) and infrared intensities (in parentheses, in km/mol) for the structure **1T−1** of $(C_4H_6)_2Fe_2(CO)$ structure.

BP86	M06-L	B3LYP	BP86	M06-L	B3LYP	BP86	M06-L	B3LYP
51(0)	23(1)	26(1)	584(14)	587(10)	563(1)	1215(2)	1254(4)	1270(1)
66(0)	34(0)	40(0)	665(0)	688(0)	657(4)	1337(0)	1375(0)	1396(2)
74(0)	70(0)	91(1)	678(2)	691(7)	681(7)	1338(0)	1376(3)	1397(0)
101(1)	101(0)	100(0)	698(0)	696(2)	735(11)	1389(6)	1428(11)	1446(23)
131(0)	108(0)	118(0)	735(7)	750(13)	760(2)	1395(4)	1433(5)	1453(7)
141(3)	141(3)	140(0)	811(7)	816(60)	829(1)	1450(31)	1488(42)	1509(42)
172(0)	155(1)	157(2)	823(1)	817(1)	850(6)	1451(20)	1494(9)	1512(23)
254(4)	225(3)	175(13)	824(11)	821(4)	852(71)	1451(5)	1495(26)	1530(31)
280(0)	280(0)	289(0)	830(54)	842(38)	861(3)	1456(9)	1501(26)	1541(17)
311(4)	300(4)	296(2)	849(0)	863(1)	892(5)	1929(857)	2006(924)	2006(1365)
355(3)	357(2)	324(5)	860(14)	878(5)	908(0)	3033(6)	3091(27)	3116(0)
372(0)	372(1)	344(4)	866(3)	881(2)	909(26)	3034(7)	3092(27)	3116(2)
388(2)	394(1)	369(4)	871(6)	888(10)	925(1)	3044(3)	3110(11)	3122(0)
413(0)	395(2)	400(0)	895(0)	916(10)	931(0)	3044(1)	3110(3)	3123(2)
437(5)	428(1)	413(6)	905(0)	927(1)	941(7)	3068(0)	3131(6)	3128(3)
445(3)	465(0)	431(3)	1001(5)	1029(5)	1051(4)	3068(4)	3132(0)	3128(9)
472(7)	490(5)	433(2)	1003(4)	1030(4)	1052(1)	3084(1)	3148(39)	3150(12)
474(0)	502(26)	476(0)	1027(1)	1061(1)	1074(1)	3085(14)	3148(2)	3150(0)
501(3)	518(2)	510(4)	1032(13)	1066(10)	1074(24)	3126(4)	3183(6)	3205(0)
518(17)	534(12)	539(34)	1156(1)	1194(13)	1195(17)	3127(8)	3185(29)	3206(4)
562(12)	556(13)	545(13)	1157(7)	1194(1)	1202(1)	3133(0)	3215(1)	3222(2)
562(6)	561(10)	556(19)	1207(20)	1246(34)	1264(42)	3133(7)	3215(19)	3222(6)

Table S40. Harmonic vibrational frequencies (in cm^{-1}) and infrared intensities (in parentheses, in km/mol) for the structure **1T−2** of $(C_4H_6)_2Fe_2(CO)$ structure.

BP86	M06-L	B3LYP	BP86	M06-L	B3LYP	BP86	M06-L	B3LYP
32(1)	59(1)	−71(0)	593(0)	666(1)	631(2)	1226(7)	1227(8)	1273(13)
64(0)	89(0)	−50(0)	668(2)	716(10)	668(5)	1352(0)	1361(1)	1406(0)
73(1)	111(0)	41(0)	669(0)	738(1)	689(0)	1358(0)	1384(0)	1410(0)
87(0)	121(2)	45(2)	682(1)	764(0)	733(0)	1418(4)	1437(3)	1472(13)
88(0)	127(2)	104(0)	686(2)	780(1)	734(14)	1420(8)	1460(1)	1480(6)
142(0)	174(0)	111(0)	831(14)	815(15)	826(14)	1453(7)	1463(7)	1520(5)
169(1)	181(1)	171(2)	839(12)	845(3)	829(29)	1457(4)	1468(5)	1523(14)
196(0)	237(12)	203(0)	850(4)	869(2)	878(0)	1457(2)	1498(5)	1526(20)
219(0)	281(3)	220(1)	869(2)	901(0)	898(7)	1465(4)	1506(5)	1528(7)
268(2)	309(10)	250(6)	874(4)	909(8)	905(7)	1835(681)	1901(736)	1874(777)
360(3)	350(35)	258(0)	880(3)	913(3)	912(3)	3044(3)	2968(0)	3137(5)
376(15)	370(0)	331(6)	902(1)	925(3)	928(4)	3046(7)	2981(9)	3137(3)
383(2)	431(5)	335(11)	903(7)	929(3)	947(3)	3052(3)	3099(38)	3138(4)
399(6)	433(11)	345(0)	907(3)	944(2)	959(0)	3054(6)	3104(27)	3139(0)
416(0)	450(20)	387(1)	907(2)	949(3)	960(1)	3064(2)	3135(8)	3144(4)
428(1)	470(0)	413(1)	1018(14)	1037(5)	1059(3)	3073(2)	3145(2)	3145(3)
439(15)	477(14)	431(5)	1020(7)	1040(12)	1066(9)	3079(15)	3151(37)	3161(10)
448(0)	484(0)	435(10)	1036(1)	1058(5)	1071(6)	3088(10)	3157(28)	3162(16)
459(1)	498(2)	450(80)	1039(7)	1068(8)	1079(13)	3134(11)	3172(46)	3223(6)
499(20)	499(24)	465(1)	1158(4)	1162(9)	1199(5)	3134(8)	3175(20)	3223(3)
556(8)	538(13)	514(16)	1160(8)	1189(5)	1208(14)	3141(5)	3191(12)	3228(3)
581(0)	625(1)	592(4)	1218(4)	1191(7)	1246(17)	3142(2)	3193(21)	3229(8)

Table S41. Harmonic vibrational frequencies (in cm^{-1}) and infrared intensities (in parentheses, in km/mol) for the structure **1Q−1** of $(C_4H_6)_2Fe_2(CO)$ structure.

BP86	M06-L	B3LYP	BP86	M06-L	B3LYP	BP86	M06-L	B3LYP
61(0)	56(0)	9(0)	581(9)	589(3)	583(13)	1226(9)	1258(11)	1274(9)
70(0)	70(0)	35(0)	664(3)	663(6)	702(0)	1340(1)	1376(2)	1397(1)
100(0)	107(0)	44(0)	691(1)	700(1)	711(1)	1345(0)	1382(1)	1397(0)
116(0)	119(0)	104(0)	701(2)	712(2)	714(24)	1388(15)	1424(14)	1445(40)
130(0)	137(1)	116(0)	719(13)	725(15)	758(20)	1403(8)	1441(7)	1454(18)
148(2)	149(2)	136(0)	807(16)	819(14)	826(0)	1444(11)	1485(22)	1506(50)
154(1)	170(1)	150(6)	824(16)	837(9)	832(92)	1452(18)	1491(11)	1513(1)
255(0)	265(0)	174(4)	833(29)	845(5)	846(12)	1461(25)	1498(27)	1522(37)
274(0)	280(0)	277(2)	834(11)	850(52)	860(19)	1477(17)	1521(21)	1532(68)
285(0)	289(1)	278(0)	857(13)	872(13)	888(1)	1925(870)	1998(957)	1994(1052)
321(2)	328(3)	312(7)	864(3)	877(1)	904(0)	3029(6)	3094(22)	3111(10)
329(3)	333(1)	334(1)	877(1)	892(1)	919(8)	3034(2)	3097(14)	3112(10)
368(2)	387(2)	356(0)	891(5)	905(7)	935(7)	3052(3)	3115(9)	3115(0)
394(3)	399(5)	376(1)	912(2)	925(5)	937(4)	3057(5)	3116(15)	3117(6)
433(4)	448(7)	403(1)	920(0)	938(1)	960(0)	3061(2)	3130(7)	3138(0)
448(3)	468(2)	438(0)	1005(3)	1029(4)	1049(5)	3068(5)	3132(17)	3138(3)
477(6)	495(2)	444(1)	1008(5)	1031(6)	1049(3)	3077(5)	3148(34)	3149(0)
485(13)	503(23)	489(45)	1036(2)	1066(2)	1074(0)	3085(9)	3149(7)	3150(13)
506(3)	525(3)	513(4)	1042(5)	1078(3)	1078(13)	3122(5)	3191(13)	3197(2)
524(12)	530(11)	544(9)	1162(8)	1197(10)	1200(16)	3124(4)	3192(11)	3199(11)
548(9)	540(12)	554(14)	1164(2)	1200(1)	1205(0)	3150(3)	3217(11)	3231(0)
556(6)	567(4)	566(11)	1212(15)	1245(20)	1264(56)	3155(2)	3228(9)	3231(2)

Table S42. Harmonic vibrational frequencies (in cm^{-1}) and infrared intensities (in parentheses, in km/mol) for the structure **1Q−2** of $(C_4H_6)_2Fe_2(CO)$ structure.

BP86	M06-L	B3LYP	BP86	M06-L	B3LYP	BP86	M06-L	B3LYP
35(0)	39(0)	33(0)	578(4)	593(6)	585(4)	1225(9)	1263(12)	1273(21)
66(1)	76(1)	54(2)	642(9)	651(18)	657(22)	1346(0)	1383(0)	1400(1)
86(0)	87(0)	76(0)	647(3)	658(1)	670(4)	1355(0)	1391(0)	1409(0)
112(1)	116(0)	99(1)	657(17)	684(3)	678(30)	1405(20)	1446(15)	1455(16)
118(0)	123(0)	109(1)	670(1)	689(3)	691(3)	1417(5)	1455(6)	1466(11)
128(0)	132(1)	117(1)	808(41)	820(53)	833(38)	1433(3)	1488(11)	1494(3)
201(0)	206(1)	187(1)	834(8)	841(7)	845(17)	1465(7)	1510(45)	1521(29)
212(2)	226(1)	198(0)	835(7)	848(4)	852(7)	1465(29)	1512(7)	1528(24)
245(1)	249(1)	238(3)	841(5)	857(9)	867(16)	1474(11)	1522(12)	1534(16)
262(0)	279(0)	251(0)	863(1)	871(3)	893(4)	1802(666)	1877(782)	1891(933)
315(3)	319(2)	273(2)	867(0)	881(3)	907(1)	3015(4)	3079(14)	3097(3)
346(2)	355(3)	307(2)	892(1)	916(2)	922(3)	3046(5)	3113(11)	3129(6)
363(8)	363(4)	319(8)	898(3)	924(4)	932(2)	3053(2)	3115(12)	3142(1)
369(2)	380(2)	347(6)	909(1)	929(2)	941(1)	3063(2)	3123(9)	3146(2)
398(3)	418(2)	381(4)	912(8)	942(12)	963(1)	3076(2)	3139(7)	3151(2)
412(1)	425(9)	401(22)	1004(5)	1039(6)	1039(4)	3077(3)	3149(10)	3155(2)
432(8)	439(10)	408(3)	1023(8)	1054(10)	1057(5)	3089(4)	3155(31)	3165(6)
440(9)	455(2)	433(4)	1038(6)	1071(5)	1079(11)	3091(13)	3163(6)	3172(10)
453(4)	469(5)	454(4)	1049(10)	1080(10)	1085(10)	3101(9)	3176(20)	3180(9)
467(14)	472(20)	458(10)	1155(6)	1197(5)	1197(12)	3146(4)	3220(12)	3227(8)
502(6)	494(7)	483(14)	1168(4)	1207(6)	1214(6)	3147(8)	3221(18)	3235(3)
569(7)	581(4)	580(8)	1209(25)	1246(31)	1254(18)	3158(4)	3230(12)	3240(5)
